# Anlotinib potentiates anti‐PD1 immunotherapy via transferrin receptor‐dependent CD8^+^ T‐cell infiltration in hepatocellular carcinoma

**DOI:** 10.1002/ctm2.1738

**Published:** 2024-08-02

**Authors:** Fei Song, Bo Hu, Xiao‐Liang Liang, Jian‐Wen Cheng, Cheng‐Gui Wang, Peng‐Xiang Wang, Tian‐Lun Wang, Peng‐Ju Tang, Hai‐Xiang Sun, Wei Guo, Jian Zhou, Jia Fan, Zhong Chen, Xin‐Rong Yang

**Affiliations:** ^1^ Department of Liver Surgery & Transplantation Liver Cancer Institute Zhongshan Hospital Fudan University; Key Laboratory of Carcinogenesis and Cancer Invasion, Ministry of Education Shanghai P. R. China; ^2^ Department of Hepatobiliary Surgery Affiliated Hospital of Nantong University Nantong P. R. China; ^3^ Department of Laboratory Medicine Zhongshan Hospital Fudan University Shanghai P. R. China; ^4^ Institutes of Biomedical Sciences Fudan University Shanghai P. R. China

**Keywords:** hepatocellular carcinoma, immune checkpoint blockade, transferrin receptor, tumour microenvironment, tyrosine kinase inhibitor

## Abstract

**Background:**

The therapeutic potential of immune checkpoint blockade (ICB) extends across various cancers; however, its effectiveness in treating hepatocellular carcinoma (HCC) is frequently curtailed by both inherent and developed resistance.

**Objective:**

This research explored the effectiveness of integrating anlotinib (a broad‐spectrum tyrosine kinase inhibitor) with programmed death‐1 (PD‐1) blockade and offers mechanistic insights into more effective strategies for treating HCC.

**Methods:**

Using patient‐derived organotypic tissue spheroids and orthotopic HCC mouse models, we assessed the effectiveness of anlotinib combined with PD‐1 blockade. The impact on the tumour immune microenvironment and underlying mechanisms were assessed using time‐of‐flight mass cytometry, RNA sequencing, and proteomics across cell lines, mouse models, and HCC patient samples.

**Results:**

The combination of anlotinib with an anti‐PD‐1 antibody enhanced the immune response against HCC in preclinical models. Anlotinib remarkably suppressed the expression of transferrin receptor (TFRC) via the VEGFR2/AKT/HIF‐1α signaling axis. CD8^+^ T‐cell infiltration into the tumour microenvironment correlated with low expression of TFRC. Anlotinib additionally increased the levels of the chemokine CXCL14, crucial for attracting CD8^+^ T cells. CXCL14 emerged as a downstream effector of TFRC, exhibiting elevated expression following the silencing of *TFRC*. Importantly, low TFRC expression was also associated with a better prognosis, enhanced sensitivity to combination therapy, and a favourable response to anti‐PD‐1 therapy in patients with HCC.

**Conclusions:**

Our findings highlight anlotinib's potential to augment the efficacy of anti‐PD‐1 immunotherapy in HCC by targeting TFRC and enhancing CXCL14‐mediated CD8^+^ T‐cell infiltration. This study contributes to developing novel therapeutic strategies for HCC, emphasizing the role of precision medicine in oncology.

**Highlights:**

- Synergistic effects of anlotinib and anti‐PD‐1 immunotherapy demonstrated in HCC preclinical models.
- Anlotinib inhibits TFRC expression via the VEGFR2/AKT/HIF‐1α pathway.
- CXCL14 upregulation via TFRC suppression boosts CD8+ T‐cell recruitment.
- TFRC emerges as a potential biomarker for evaluating prognosis and predicting response to anti‐PD‐1‐based therapies in advanced HCC patients.

## Graphical abstract

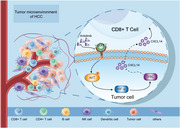

## 1 INTRODUCTION

The advancement in immune checkpoint blockade (ICB) therapies has transformed the clinical approach to treating a wide array of cancers, notably hepatocellular carcinoma (HCC).^1^, ^2^, ^3^ Nonetheless, the overall response rate of HCC to PD‐1/PD‐L1 antibodies remains unsatisfactory (pembrolizumanb, 18.3%; nivolumab, 15.0%; camrelizumab, 14.7%), and few patients achieve complete remission due to primary or acquired ICB resistance.^4^, ^5^, ^6^ Thus, it is critically important to explore novel combinatory treatment approaches.

Recent research has demonstrated that the concurrent use of a tyrosine kinase inhibitor (TKI) and anti‐PD‐1 antibodies can lead to enhanced synergistic effects in tumour suppression.^7^, ^8^, ^9^, ^10^ Anlotinib, a multi‐targeted tyrosine kinase inhibitor, demonstrates extensive anti‐tumour efficacy across various cancer types. It primarily targets vascular endothelial growth factor receptors, fibroblast growth factor receptors, and platelet‐derived growth factor receptors (PDGFRα and PDGFRβ), among others. By inhibiting these receptors, anlotinib disrupts tumour angiogenesis, which is critical for tumour growth and metastasis.^11^ Anlotinib promoted the normalization of blood vessels and enhanced the entry of immune cells, thereby altering the tumour microenvironment (TME).^12^ Recent studies from clinical trials suggest that combining anlotinib with sintilimab may offer an effective secondary or subsequent treatment option for individuals battling advanced cervical cancer.^13^ Enhanced therapeutic effectiveness was also noted in a preliminary phase study evaluating the synergistic impact of anlotinib combined with anti‐PD‐1 therapy.^14^ This evidence indicates that the concurrent use of anlotinib and an anti‐PD‐1 antibody could result in synergistic tumour suppression. Nevertheless, the potential mechanism underlying this enhanced efficacy of anti‐PD‐1 therapy in HCC remains inadequately defined.

In this study, we investigated the combined impact of anlotinib and an anti‐PD‐1 antibody through the use of patient‐derived organotypic tumour spheroids (PDOTs) and a preclinical mouse model for HCC. To delve into the potential mechanisms behind the observed shifts in efficacy, we conducted a time‐of‐flight mass cytometry (CyTOF) analysis on orthotopic tumours derived from mouse models of HCC. Finally, we confirmed our findings using tissue samples and clinical data from patients with HCC.

## 2 MATERIALS AND METHODS

### 2.1 Patients and specimens

We procured a cohort of 24 microfluidic PDOTs derived from primary HCC biopsies from Zhongshan Hospital, Fudan University. None of the patients in this cohort (*n* = 24) had received chemotherapy, radiotherapy, or any other anti‐tumour therapy prior to the collection of their biopsies. Detailed clinical characteristics of these treatment‐naive patients are presented in Table S1.

We collected biopsies from 21 patients with unresectable HCC who were being treated with PD‐1 immunotherapy. These samples were used to assess whether there was a correlation between TFRC and drug responsiveness using immunohistochemistry.^15^ Subsequently, an independent radiology review was carried out to obtain a radiographic evaluation of the response. The assessment of tumour responses in these patients was guided by the mRECIST criteria.^16^ A detailed description of the clinical characteristics of these patients is provided in Table S2.

The Research Ethics Committee of Zhongshan Hospital, Fudan University governed both studies. Neither of these studies was conducted without written informed consent from patients. All participants provided written informed consent for the collection and use of their tissue samples (approval no. B2021‐689R).

### 2.2 Three‐dimensional culture system of patient‐derived organotypic tissue spheroids using a microfluidic chip

Detailed procedures for PDOTS model construction^17^ are described in the Supporting information Materials and Methods section. Following exposure to the treatments for 5−7 days, the culture medium was carefully removed, and PDOTs underwent staining using 10 μL of AO/PI solution (Nexcelom Bioscience) in dark conditions at 4°C for 5 min.^18^ Using fluorescence microscopy, live (green) and dead (red) cells were distinguished. Image J software facilitated the quantification of cell viability, gauging the balance between red and green fluorescence intensities. The tumour‐killing index (TK‐index) was deduced as

$$\mathrm{Alive}=\;\frac{AG}{AG+AR},$$

$$\Delta \mathrm{Alive}=\mathrm{Aliv}e_{NC}-\mathrm{Aliv}e_{T},$$

$$\;TKI=\frac{\Delta \mathrm{Alive}}{\mathrm{Alive}NC}\times 100\%,$$

where AG and AR represent areas of green and red fluorescence, respectively. Meanwhile, Alive_NC_ and Alive_T_ denote cell viability in control and drug‐treated groups, respectively.

### 2.3 Animal studies

Mice with wild‐type C57BL/6 and BALB/C genotypes were purchased from Shanghai SALC and bred at Shanghai Experimental Animal Center. Male mice (aged 5 weeks) were used for all experiments. H22 cells originated from a BALB/C mouse, while hepa1‐6 cells originated from a C57BL/6 mouse. Hepa1‐6 cells (1 × 10^6^) or H22 cells were subcutaneously injected into a suitable inguinal fold region in each mouse. To establish an orthotopic HCC model, subcutaneous tumours were allowed to grow for 14 days before being removed. The orthotopic tumour mouse model was established as described previously.^19^ After six days of acclimatization, mice were randomly allocated into four treatment groups: anlotinib alone (1.5 mg/kg/day, orally administered, dissolved in .9% saline), anti‐PD‐1 alone (200 μg/mouse, administered via intraperitoneal injection every 3 days), a combination therapy group receiving both anlotinib and anti‐PD‐1 at the same dosages and schedule, and a control group receiving normal saline (.9% saline solution, matching the vehicle for anlotinib). Treatments were administered for 12 days. The tumours were then removed, weighed, and minced for further analyses after the mice were sacrificed. Following the schedule shown in Figure 3A, mice were administered anti‐CD8a, anti‐CD4, or both anti‐CD8a and anti‐CD4 antibodies to deplete T cells in vivo. All tests were carried out with the goal of minimizing the number of mice used and alleviating their distress to the greatest extent possible. Studies using anesthesia or euthanasia methods adhered to veterinary best practices. A liver with tumours was removed for examination after mice were euthanized following the prescribed treatment(s). The tumour volume (mm^3^) was calculated using the formula: length (mm) × width^2^ (mm) × .5. All mouse experiments were performed following protocols approved by the Institutional Animal Care and Use Committee of Fudan University. Additional information on methods is available in Supporting Information Data.

## 3 RESULTS

### 3.1 Anlotinib improves the anti‐tumour activity of PD‐1 blockade in vitro and in vivo

**FIGURE 1:**
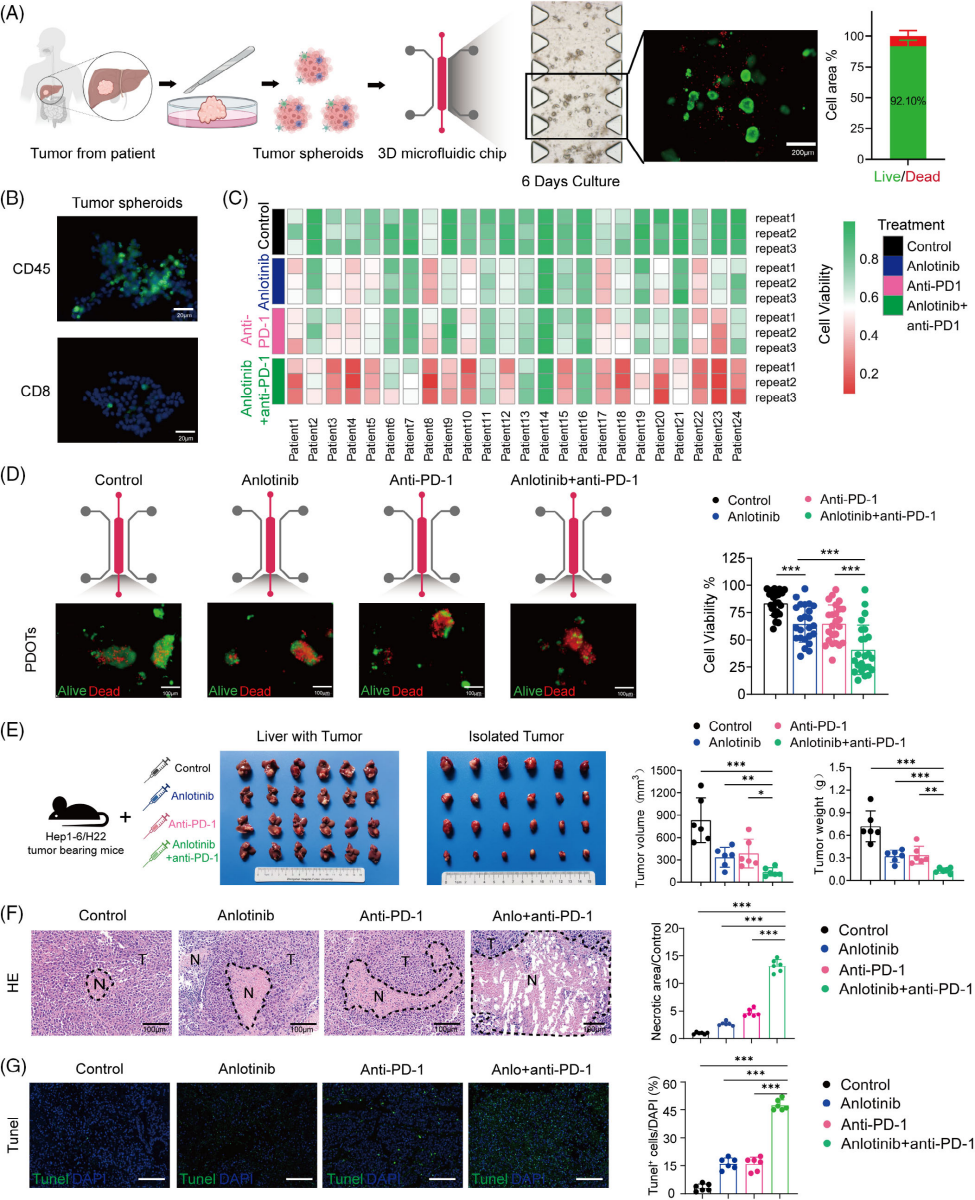
Anlotinib improves the anti‐tumour activity of PD‐1 blockade in vitro and vivo. (A) Schematic showing the preparation of HCC patient‐derived PDOTs (40–100 μm diameter). Viability assessment of PDOTs cultured in a microfluidic chip for 7 days using fluorescent staining (live = green, dead = red). Scale bars: 200 μm. (B) Immunofluorescent staining of CD45^+^ (green) and CD8^+^ (green) immune cells within PDOTs. Scale bars: 25 μm. (C) Heat map illustrating the viability of PDOTs cultured under different treatments: control (DMSO+IgG), anlotinib (10 μmol), anti‐PD‐1 (250 μg/mL), and combined anlotinib+anti‐PD‐1. (D) Representative image and quantitative analysis of the viability of PDOTs (*n* = 24) after different treatments as indicated by staining for acridine orange (AO, live cells shown in green) and propidium iodide (PI, dead cells shown in red). Scale bars: 100 μm; (E) Representative images of orthotopic Hep1‐6 tumours in C57BL/6 mice after various treatments (*n* = 6 per group), accompanied by graphs of tumour volumes (left) and weights (right) for the depicted tumours. (F, G) Representative H&E and Tunel images of the primary tumours from C57BL/6 mice. Scale bar: 100 μm. T, tumour; N, necrosis. Data are presented as mean ± SD, *p < 0.05; **p < 0.01; ***p < 0.001.

To assess the efficacy of anti‐PD‐1 treatment alone or combined with anlotinib in vitro, we used a 3D microfluidic device (AIM Biotech, DAX‐1 idenTx 3 Chip) to develop a short‐term culture of HCC PDOTs.^17^, ^18^ The construction of the PDOTs model was streamlined to essential steps (Figure 1A), with complete protocol details provided in the Supporting Information Materials and Methods section. The immune cells in the PDOTs were further confirmed by immunofluorescence microscopy and flow cytometry (Figure 1B and Figure S1). The PDOTs were loaded in collagen into the central channel of a 3D microfluidic device, and were stained after six days in 3D microfluidic culture by AO/PI, which indicated that up to 92.01 ± 2.71% of the PDOTs remained viable for at least one week in culture (Figure 1A). The PDOTs derived from 24 HCC patients were cultured in collagen in the central channel of the 3D microfluidic device and then treated with anlotinib and/or anti–PD‐1 antibodies (sintilimab). The synergy between anlotinib and an anti‐PD‐1 antibody significantly enhanced tumour suppression, surpassing the effects seen with either anlotinib or anti‐PD‐1 therapy alone (anti‐PD‐1 vs. Anlotinib+Anti‐PD‐1, 64.84 ± 17.31 vs. 40.93 ± 22.64, *p* < 0.001; Anlotinib vs. Anlotinib+anti‐PD‐1, 64.56 ± 16.87 vs. 40.93 ± 22.64, *p* < 0.001) (Figure 1C, D). To further elucidate whether the anti‐tumour effects elicited by the combination therapy were synergistic or additive, we utilized the widely recognized combination drug index (CDI) method to assess drug interactions.^20^ The CDI is calculated using the formula $\mathrm{CDI}=\frac{\mathrm{AP}}{A\times P}$, where “*A*” denotes the average activity rate of the anlotinib group, “*P*” represents the average activity rate of the anti‐PD1 group, and “AP” refers to the average activity rate of the combination therapy group. Our findings revealed that the average activity rate was 74.98% for the anlotinib group, 75.87% for the anti‐PD1 group, and 56.09% for the combination therapy group, yielding a CDI of .98. This CDI value, being less than 1, indicates a synergistic effect, thereby validating the significant synergistic enhancement offered by our combination therapy.

We then examined the efficacy of combination treatment in mouse models of HCC (C57BL/6 and BALB/c mice; Figure 1E). While anlotinib and anti‐PD‐1 independently yielded significant tumour growth reduction in HCC models, the combined regimen demonstrated more potent inhibition of tumour growth, as evidenced by measurements of tumour volume and weight (Figure 1E and Figure S2A, B). The efficacy of the combination therapy was quantified using the CDI value, calculated to be .89, which indicates the synergistic effects of anlotinib and anti‐PD‐1. Specifically, the average tumour volume in the anlotinib group was 40.23% of the control group, in the anti‐PD‐1 group it was 46.26%, and in the combination therapy group, it was significantly reduced to 16.55%. These results underscore the synergistic efficacy of the combination treatment in vivo. Moreover, preliminary observations from our animal studies indicate that the combination therapy of anlotinib and anti‐PD‐1 immunotherapy maintains the general well‐being of the subjects, without significant weight loss or adverse changes in feeding behaviour (Figure S2C).

Upon completion of the treatment, tumour specimens were harvested for additional study. Both the anlotinib and anti‐PD‐1 monotherapy groups exhibited increased tumour necrosis relative to the control group, yet the most substantial necrosis was observed in the combined treatment group across both models (Figure 1F and Figure S2D, E; all *p*‐values < 0.001). Additionally, TUNEL staining indicated a significant increase in apoptotic cells in both monotherapy groups compared with the control. The combination therapy group demonstrated the highest percentage of TUNEL‐positive cells, suggesting a synergistic effect on inducing tumour cell apoptosis (Figure 1G, right bar graph; *p* < 0.001). No statistically significant variances were observed in body weight across the groups, indicating an absence of overt toxicity in any of the treatment cohorts (Figure S2C; all *p*‐values > 0.05). Collectively, our findings demonstrate that anlotinib can amplify the anti‐tumour effects of PD‐1 blockade. This aligns with the outcomes from phase Ib/II trials of anlotinib and penpulimab combination therapy, which showed tolerable toxicity and promising clinical efficacy in patients with advanced HCC.^21^

### 3.2 CD8^+^ T‐cell infiltration is increased after PD‐1 blockade when used in combination with anlotinib treatment

**FIGURE 2:**
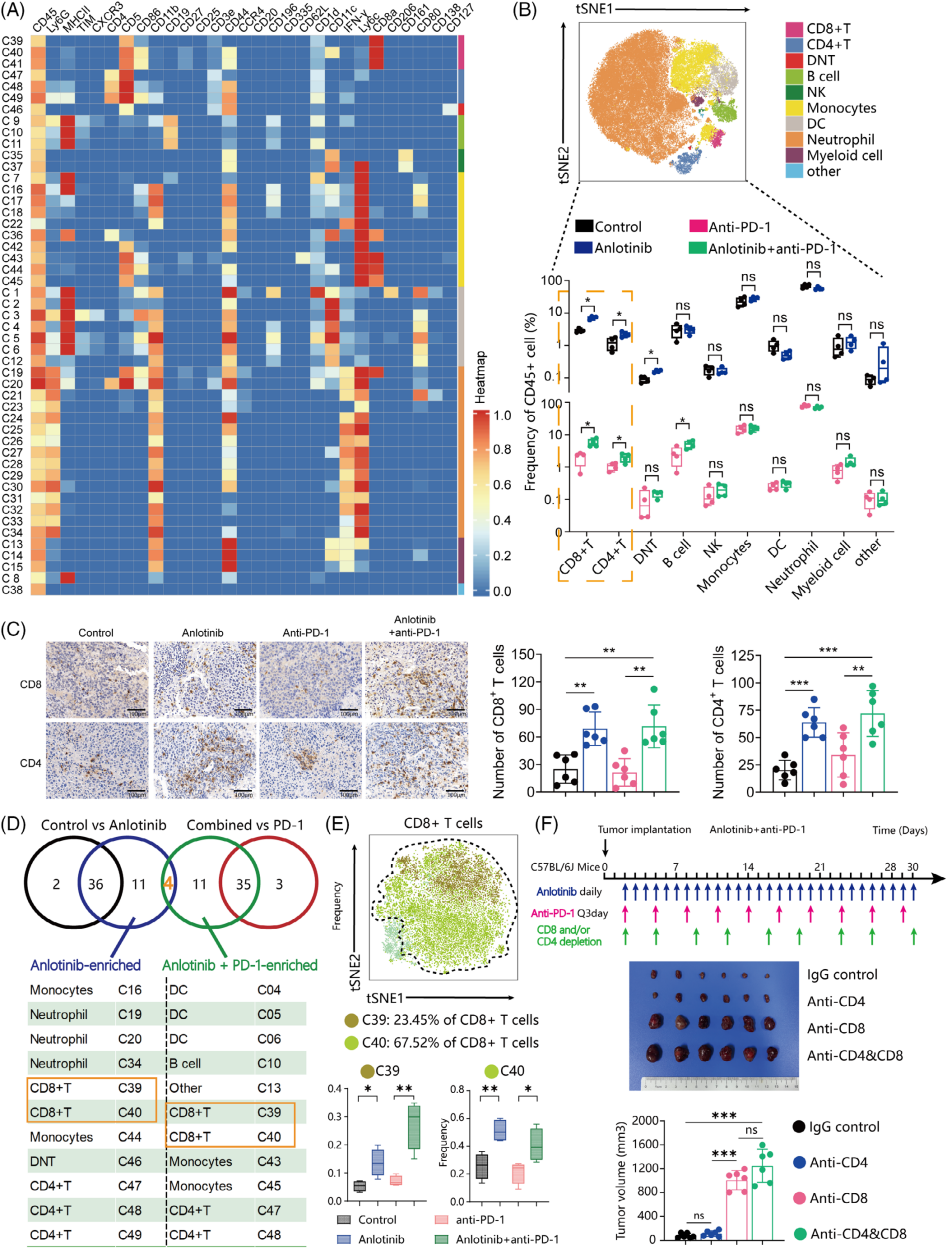
Anlotinib plays a crucial role in the regulation of tumour immunity. (A) Heatmap of selected immune markers for the 49 clusters in the CyTOF data. (B) tSNE plots of immune cell subpopulations. Box plots of differential immune cell subpopulations in each indicated group. (C) Representative images and bar plots illustrating the number of CD8^+^ and CD4^+^ T cells in Hepa1‐6 tumours from mice that received the indicated treatments, Scale bar: 100 μm. (D) Venn diagram of enriched cell subpopulations within each indicated group. (E) t‐SNE plot of the CD8^+^ T‐cell subpopulation distribution. Boxplot illustrating the fractions of cells in clusters 39 and 40 within each indicated group. (F) Schedule of tumour implantation and injection of antibodies for cell depletion in mice. Depletion of CD8^+^ or CD4^+^ cells during combination therapy with anlotinib and anti‐PD‐1. Bar plot showing the tumour size in each group (*n* = 6) following CD8^+^ and/or CD4^+^ T‐cell depletion. Data are presented as mean ± SD, *p < 0.05; **p < 0.01; ***p < 0.001; ns, not significant.

We employed CyTOF for the examination of tumour specimens across different treatment cohorts within the C57BL/6 model. Twenty‐nine common immune markers were examined, allowing the immune cells to be categorized into 49 clusters (Figure 2A). Based on the similarity of marker expression between different immune cell clusters, an unsupervised clustering method was utilized to group these clusters into 10 immune cell populations (Figure 2B). A notable enhancement in the populations of both CD8^+^ and CD4^+^ T cells was observed in the anlotinib‐treated group relative to the control group. The group receiving combination therapy (Anlotinib+anti‐PD‐1) showed a substantial rise in T‐cell counts compared with the cohort treated solely with anti‐PD‐1 (Figure 2B). Subsequently, we employed immunohistochemistry to validate the infiltration of CD8^+^ T cells and CD4^+^ T cells in the various groups. There was a significant increase in infiltrating CD8^+^ and CD4^+^ T cells in the anlotinib group compared with the control group (Figure 2C and Figure S3A, B; all *p*‐values < 0.01). Likewise, the combined treatment group (Anlotinib + anti‐PD‐1) demonstrated a marked elevation in T‐cell levels in comparison to the anti‐PD‐1‐only group (Figure 2C and Figure S3A, B; all *P*‐values < 0.01).

To identify which immune cell populations were contributing to the improved efficacy, we examined the distribution of immune cell subgroups. The anlotinib monotherapy group exhibited enrichment in 11 immune cell subclusters, encompassing CD8^+^ T cells (C39, C40), CD4^+^ T cells (C47, C48, C49), monocytes (C16, C44), and neutrophils (C19, C20, C34). There were also 11 enriched immune cells subclusters in the combined treatment group, including CD8^+^ T cells (C39, C40), CD4^+^ T cells (C47, C48), dendritic cells (C4, C5, C6), B cells (C10), and monocytes (C43, C45). We found that C39 and C40 (CD8^+^ T cells), and C47 and C48 (CD4^+^ T cells), were enriched in both the anlotinib and combined treatment groups (Figure 2D). Notably, clusters C39 (CD86^−^CD44^−^CD11c^−^) and C40 (CD86^+^CD44^+^CD11c^+^) represented 91.0% of all CD8^+^ T cells (Figure 2E), while Cluster 47 (CD4^+^CD5^+^CD44^−^) and Cluster 48 (CD4^+^CD5^+^CD86^+^) comprised 18.62% of all CD4^+^ T cells (Figure S3C).

To determine the critical role of CD8^+^ and/or CD4^+^ T cells in the sensitization effect of anlotinib to anti‐PD‐1 therapy, depletion studies were carried out using anti‐CD4 and anti‐CD8 neutralizing antibodies, alongside isotype control IgG (Figure 2F). Our results indicated that the removal of CD8^+^ T cells or both CD4^+^ and CD8^+^ T cells entirely negated the tumour regression triggered by the combination therapy, while the elimination of only CD4^+^ T cells did not have a notable effect (Figure 2F). These findings suggest that the concurrent treatment with anlotinib and an anti‐PD‐1 antibody can suppress tumour cell proliferation in a manner reliant on CD8^+^ T cells.

### 3.3 Anlotinib induces immune signalling and fosters proinflammatory cytokine production

**FIGURE 3:**
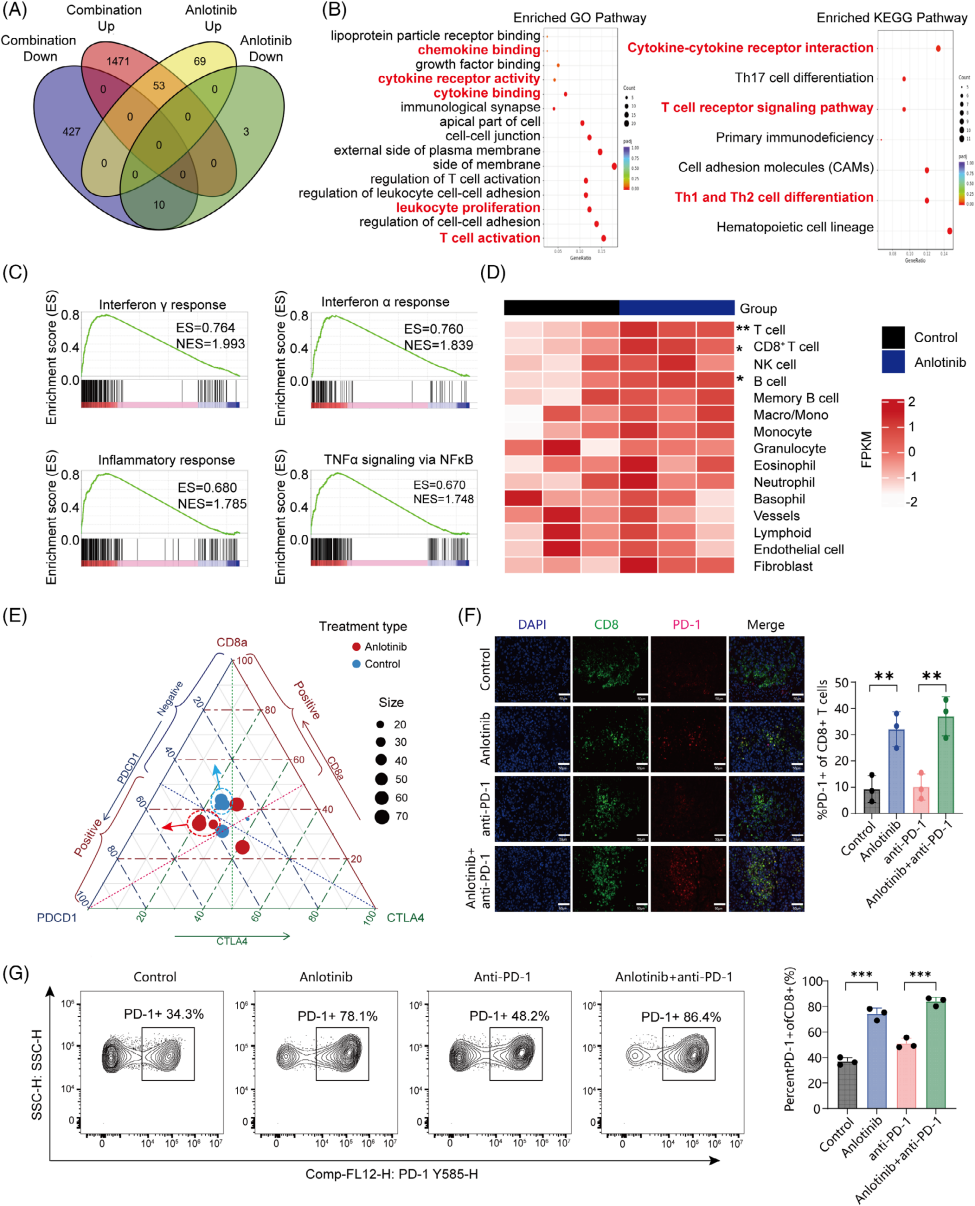
Anlotinib triggers immune response signalling and induces the production of proinflammatory cytokines and chemokines. (A) Venn diagram revealing the common up/downregulated genes (adjusted *p‐*value < 0.05, fold‐change ≥ 2) detected using RNA‐seq in groups treated with anlotinib; 63 genes were shared among the groups (53 genes upregulated, 10 genes downregulated). (B) Results of GO and KEGG enrichment analyses based on these differentially expressed genes after treatment with anlotinib versus control. (C) GSEA analysis of the differentially expressed genes induced by anlotinib monotherapy. Four of the top ten most positively regulated “Hallmark signatures”. (D) A heatmap of the degree of immune cell infiltration in the anlotinib monotherapy group as indicated by mMCP‐counter algorithms. (E) Ternary plot displaying the distribution of Hepa1‐6 tumour samples with varying proportions of PDCD1, CD8, and CTLA‐4 expression, where each axis represents the percentage (0–100%) of the respective marker. Different coloured dots represent individual tumour samples, with red indicating the anlotinib group and blue representing the control group. (F) Representative images of multiplex immunofluorescence results for Hepa1‐6 tumour tissues stained for CD8 (green), PD‐1 (red), and nuclei (DAPI; blue) and quantification of PD‐1^+^CD8^+^ T cells in mice bearing Hepa1‐6 tumours from the indicated treatment groups. (G) Representative images of multiplex immunofluorescence results for Hepa1‐6 tumour tissues stained for CD8 (green), PD‐1 (red), and nuclei (DAPI; blue) and quantification of PD‐1^+^CD8^+^ T cells in mice bearing Hepa1‐6 tumours from the indicated treatment groups. Scale bar: 50 μm. Data are presented as mean ± SD, *p < 0.05; **p < 0.01; ***p < 0.001; ns, not significant.

We then conducted a comprehensive transcriptomic analysis on tumours derived from the HCC mouse models. Our analysis identified a unique gene expression pattern in the anlotinib‐treated group versus the control group, highlighting 122 differentially expressed genes (DEGs) with increased expression and 13 DEGs with decreased expression (adjusted *p‐*value < 0.05, |fold‐change| ≥ 2.0, Figure 3A). Additionally, contrasting the combination therapy group with the anti‐PD‐1 monotherapy group revealed 1524 upregulated and 437 downregulated DEGs, indicating a significant change in gene expression profiles resulting from the combination treatment (adjusted *p*‐value < 0.05, |fold‐change| ≥ 2.0, Figure 3A).

The upregulated DEGs (*n* = 122) in anlotinib‐treated tumours were subjected to GO and KEGG analyses,^22^ which revealed that there was enrichment of immune regulatory pathways, including pathways related to T‐cell activation, T‐cell receptor signalling, and cytokine‐cytokine receptor interactions (Figure 3B). Gene set enrichment analysis (GSEA) on the DEGs showed that among the top ten most significantly upregulated “Hallmark signatures”, pathways including the interferon‐γ response, interferon‐α response, inflammatory response, and tumour necrosis factor‐α signalling were prominent (Figure 3C). The upregulated DEGs in the combination treatment group showed similar results (Figure S4A, B). All of these signalling pathways are involved in immune and inflammatory responses and play essential roles in the response to anti‐tumour therapy.^23^, ^24^, ^25^

Next, we employed two different immunoinformatic algorithms (mMCP‐counter and ssGSEA) to evaluate the changes in the TME among the different groups. Consistent with the CyTOF studies, we observed a substantial increase in genes related to the infiltration of CD8^+^ T cells following anlotinib treatment (Figure 3D). Moreover, the immune and microenvironment scores showed significant enhancement in the groups treated with anlotinib (all *p‐*values < 0.05, Figure S4C).

The transcriptional changes in immune checkpoint markers, such as TIGIT, PDCD‐1, LAG3, CTLA4, CD274, and TIM3, were explored across the various treatment groups (Figure S4D, E). Only TIGIT was significantly downregulated in the combination therapy group compared with the anti‐PD‐1 monotherapy group, while none of the other checkpoint markers showed significant changes (Figure S4D, E).

As noted above, our findings indicate that the sensitization of HCC to the anti‐PD‐1 antibody by anlotinib predominantly relies on the infiltration of CD8^+^ T cells (Figure 2F). We therefore investigated the expression of PD‐1 and CTLA4, two prevalent ICB markers in CD8^+^ T cells. We extracted information related to the three‐dimensional localization of PD‐1, CTLA4, and CD8a from our bulk sequencing data. Ternary plot visualization was applied to infer the distribution pattern of immune checkpoint positivity within CD8^+^ T cells (Figure 3E). For the analysis, we classified samples with expression levels exceeding the 50% median threshold as positive. Compared with the control group, the samples from the anlotinib group demonstrated a shift towards an enrichment on the “PD‐1^+^/CD8^+^” side of the scale. Multi‐colour immunofluorescence studies verified a notable enhancement in PD‐1 expression on CD8^+^ T cells following treatment with anlotinib (NC vs. Anlotinib, *p* < 0.01; anti‐PD‐1 *vs*. Anlotinib + anti‐PD‐1, *p* < 0.01; Figure 3F). Subsequently, we conducted flow cytometric analyses on murine tumour tissues, which further confirmed that after treatment with anlotinib, there was a significant increase in the proportion of PD‐1 positive expression among CD8^+^ T cells (Anlotinib vs. NC, *p* < 0.01; Anlotinib + anti‐PD‐1 vs. anti‐PD‐1, *p* < 0.01; Figure 3G). These results indicate that the CD8^+^ T cells which infiltrated in the TME following anlotinib treatment exhibited a propensity to co‐express PD‐1. In brief, our study demonstrates that anlotinib can activate signaling pathways related to the immune response within the TME, enhancing the infiltration of CD8^+^ T cells by secreting pro‐inflammatory cytokines, especially those positive for PD‐1, ultimately intensifying the immune response to anti‐PD‐1 antibodies.

### 3.4 Anlotinib targets TFRC to regulate the immune response and inflammation via the VEGFR2/AKT/HIF‐1α axis

Sixty‐three genes that were differentially expressed across both the anlotinib monotherapy and combination therapy groups were identified, termed as co‐expressed DEGs. Among these, 53 were upregulated, including C‐X‐C motif chemokine ligand 14 (CXCL14), surfactant protein D, and hepcidin antimicrobial peptide, while 10 genes were downregulated, including the transferrin receptor (TFRC), Annexin A11 (ANXA11), and transmembrane 9 superfamily member 5 (TM9SF5). In addition, the expression of HIF‐1α was decreased to approximately 46.7% of the control level in the anlotinib monotherapy group. Similarly, the expression level of HIF‐1α in the combination therapy group was reduced to about 66.6% compared with the anti‐PD‐1 group (Figure S4F).

**FIGURE 4:**
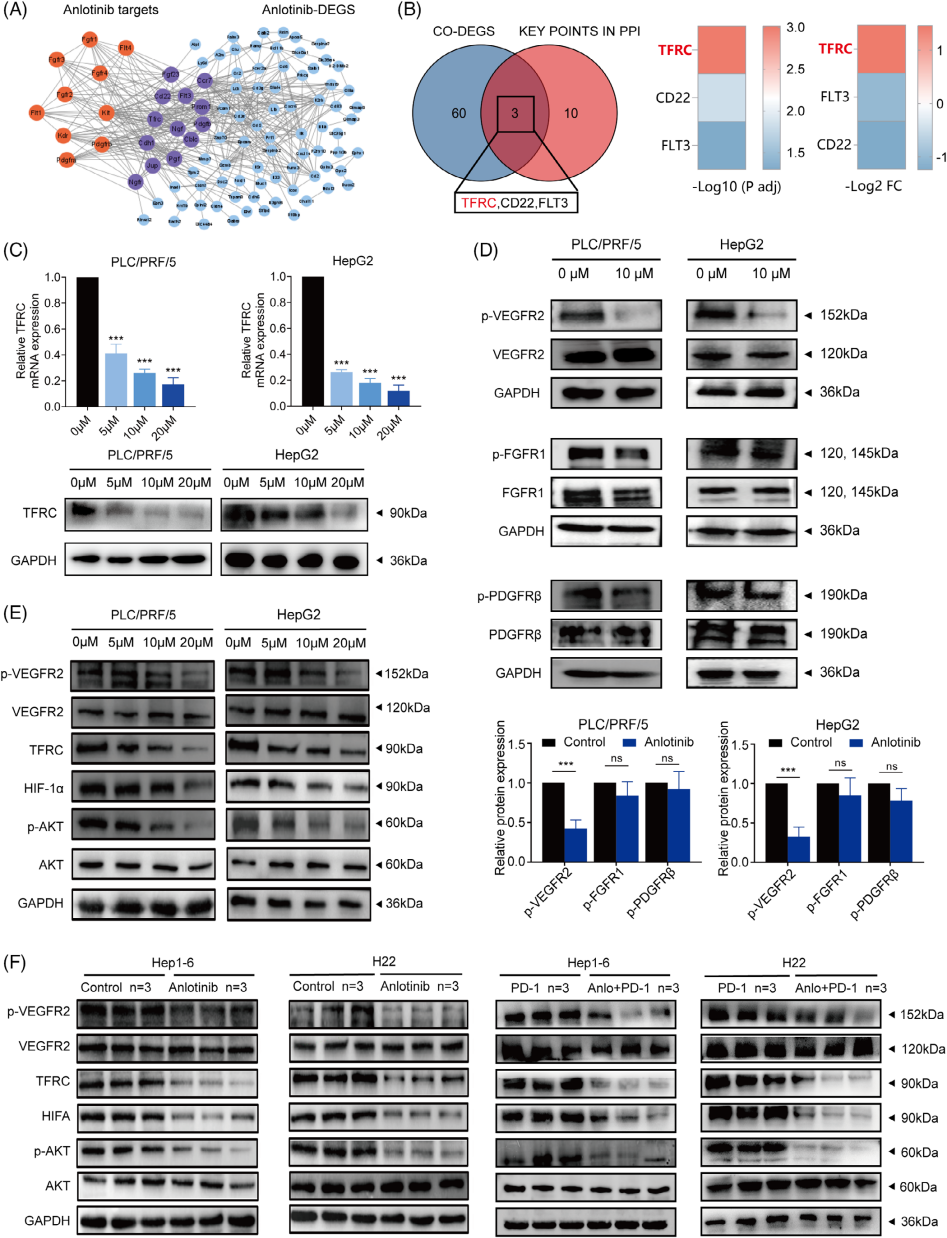
Anlotinib targets TFRC to regulate the immune response and inflammation via the VEGFR2/AKT/HIF‐1α Axis. (A) The PPI network of DEGs associated with anlotinib monotherapy vs. control group. (B) Venn diagram representing a number of overlapping differentially expressed target genes in anlotinib‐treated and combination (Anlotinib + anti‐PD‐1)‐treated tumours. Bar diagram of three common differentially expressed target genes between the anlotinib treatment and control groups. (C) qRT‐PCR and Western blot analyses of the TFRC expression levels in PLC/PRF/5 and HepG2 cells treated with increasing concentrations of anlotinib (0–20 μmol) for 24 h. (D) Western blot analysis of VEGFR2, p‐VEGFR2, FGFR1, p‐FGFR1, PDGFR‐β, and p‐PDGFR‐β expression in PLC/PRF/5 and HepG2 cell lines treated with anlotinib gradients for 24 h, with densitometry analysis from three representative experiments. (E) The expression and phosphorylation levels of VEGFR2, AKT, HIF‐1α, and TFRC in the PLC/PRF/5 and HepG2 cell lines after treatment with a concentration gradient anlotinib were detected by Western blot assays. (F) The expression and phosphorylation levels of VEGFR2, AKT, HIF‐1α, and TFRC in H22 and Hepa1‐6 tumours following the indicated treatments were detected using the Western blot assays. Data are presented as mean ± SD, *p < 0.05; **p < 0.01; ***p < 0.001; ns, not significant.

We next utilized a protein–protein interaction network and identified 13 essential targeted genes from the anlotinib‐blocked targets and 63 DEGs in tumour samples from mice with orthotopic HCC (Figure 4A). By mapping the 63 DEGs with the 13 essential targeted genes, we eventually identified three hub genes: TFRC, CD22, and Fms‐related receptor tyrosine kinase 3. Notably, TFRC showed the most significant differences in terms of both the *p*‐value and fold change in expression (Figure 4B).

We then conducted qRT‐PCR to evaluate the mRNA expression of these three hub genes. We observed that TFRC was the only gene that demonstrated concentration‐dependent downregulation in both the HepG2 and PLC/PRF/5 cell lines following treatment with anlotinib. This was particularly evident at the lowest anlotinib concentration (5 μM), where TFRC alone showed the most significant changes in both cell lines after 48 h of treatment (Figure 4C and Figure S5). Western blot analysis verified that TFRC protein expression was downregulated following anlotinib administration, occurring in a concentration‐dependent manner (Figure 4C).

TFRC transcription is predominantly regulated by the HIF‐1α signalling pathway, which is regulated by the PI3K/AKT pathway, and thus ultimately via tyrosine kinase receptors.^26^, ^27^ Interestingly, there were no significant differences in the protein levels of VEGFR2, FGFR1, or PDGFR‐β between the anlotinib‐treated and control groups, but the phosphorylation levels of VEGFR2 were significantly decreased in the anlotinib group (Figure 4D). These results imply that the TFRC inhibition induced by anlotinib might occur through its selectively blocking VEGFR2 phosphorylation (Figure 4D).

To examine this possibility, we analyzed the expression levels of critical proteins in the VEGFR2/PI3K/AKT and HIF‐1α pathways, including VEGFR2, phosphorylated VEGFR2, AKT, phosphorylated AKT, HIF‐1α, and TFRC via immunoblotting. Our observations revealed that the phosphorylation levels of VEGFR2 and AKT were significantly reduced in the anlotinib‐treated group relative to the control group (all *p*‐values < 0.05, Figure 4E, Figure S6A). Expression levels of the proteins HIF‐1α and TFRC were significantly diminished in the anlotinib group in comparison to those observed in the control group (all *p*‐values < 0.05, Figure 4E, Figure S6B). Similar results were observed in the tumour tissues of mice treated with anlotinib alone or in combination with anti‐PD‐1 (all *p*‐values < 0.05, Figure 4F, Figure S6C), indicating that anlotinib inhibits TFRC through inactivation of the VEGFR2/AKT/HIF‐1α axis.

### 3.5 The induction of CXCL14 secretion following TFRC inhibition promotes the infiltration of CD8^+^ T‐cells in the local TME

**FIGURE 5:**
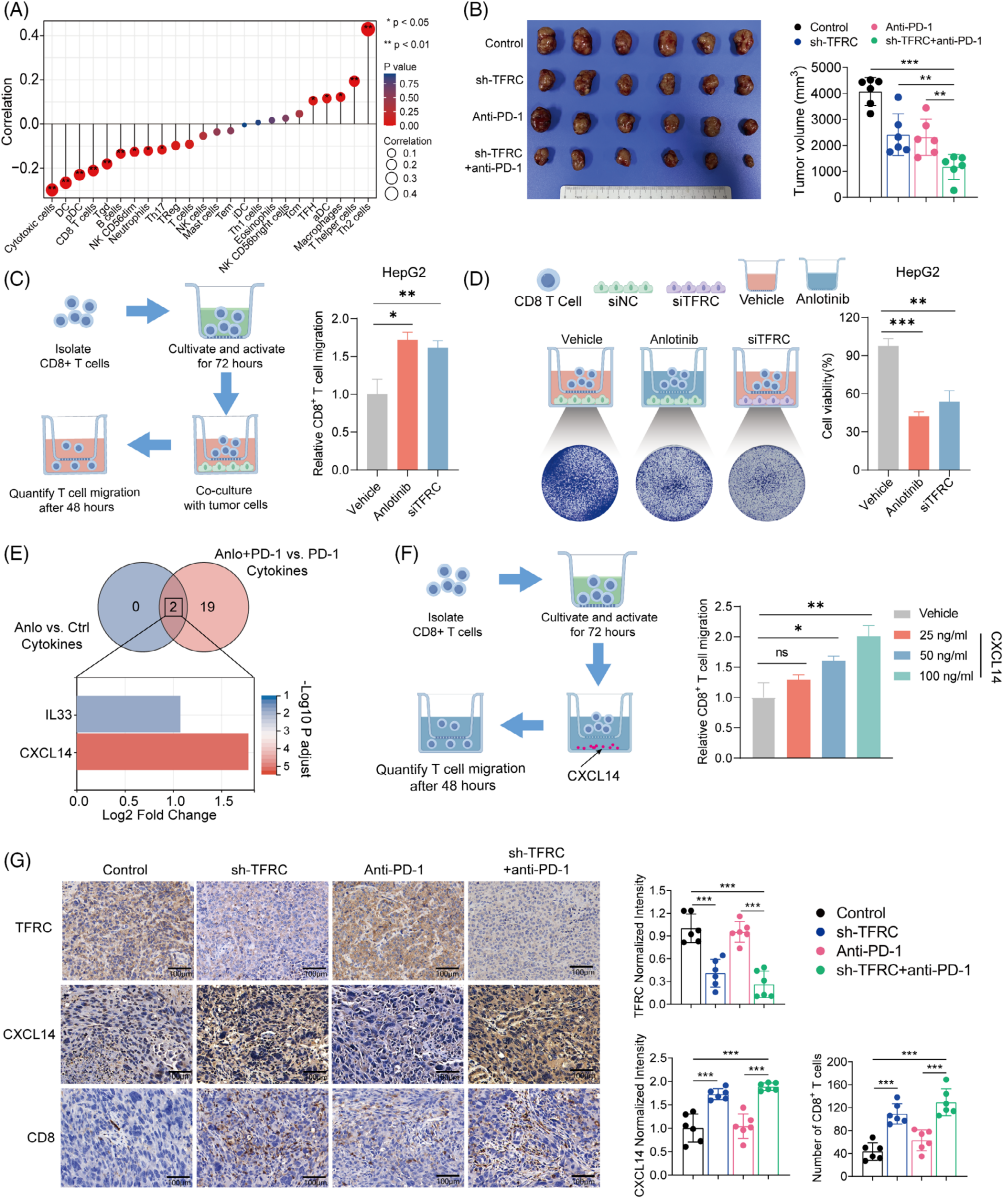
CXCL14 facilitates the infiltration of CD8^+^ T cells via inhibition of TFRC expression by Anlotinib. (A) Correlation of the TFRC mRNA levels with 24 types of immune cells. (B) Representative images and tumour volumes of TFRC wild type or TFRC‐KO tumours from C57BL/C mice from the indicated treatment groups (*n* = 6 mice per group). (C–D) Schematic representation and quantitative analysis of CD8^+^ T‐cell migration towards tumour cells and the subsequent tumour cell viability assay. Activated CD8^+^ T cells were placed in the upper chamber of a migration setup, with HepG2 tumour cells treated with either vehicle, TFRC knockdown, or anlotinib in the lower chamber. After a 48 h co‐culture period, the migrated CD8^+^ T cells were quantified. Tumour cell viability was assessed by subjecting the HepG2 cells in the lower chamber to crystal violet staining. (E) Venn diagram representing a number of overlapping differentially expressed target genes in anlotinib‐treated and combination (Anlotinib + anti‐PD‐1)‐treated tumours. (F) Schematic representation and quantitative analysis of the effects of CXCL14 on CD8^+^ T‐cell migration. Activated CD8^+^ T cells were placed in the upper chamber, with the lower chamber containing varying concentrations of CXCL14 to assess its chemotactic effect on T cells. The bar graph indicates the quantity of CD8^+^ T cells present in the lower chamber medium after a 48‐h incubation. Bar diagram of three differentially expressed cytokines that were common between the anlotinib treatment and control groups. (G) Representative immunohistochemical staining of TFRC protein expression, CXCL14 secretion and CD8^+^ T cells in TFRC wild type or TFRC‐KO tumours from C57BL/C mice following exposure to the indicated treatments, Scale bar: 100 μm. Data are presented as mean ± SD, *p < 0.05; **p < 0.01; ***p < 0.001; ns, not significant.

We hypothesized that TFRC may act as a critical mediator to promote the recruitment of CD8^+^ T‐cells in the local TME. By estimating the relative abundance of immune cell populations using the ssGSEA algorithm based on the TCGA database, we found that low TFRC expression was positively associated with increased infiltration of cytotoxic CD8^+^ T cells into tumour tissues (Figure 5A and Figure S7A). We developed a mouse tumour model with *TFRC* knockdown utilizing a lentiviral vector to transduce short hairpin RNA (shRNA) targeting the *TFRC* gene into the Hepa1‐6 cell line. The qRT‐PCR and immunoblot assays confirmed the effectiveness of the *TFRC* knockdown in these cells (Figure S7B, C). The *TFRC* downregulation significantly boosted the effects of the anti‐PD‐1 antibody in vivo (Figure 5B). Tumour volumes were markedly reduced in mice with shTFRC‐modified tumours receiving anti‐PD‐1 antibody treatment, compared with those in mice with parental Hepa1‐6 cell tumours also treated with the anti‐PD‐1 antibody (1171.13 ± 477.89 mm^3^ vs. 2314.61 ± 699.20 mm^3^, *p* = 0.008, Figure 5B).

Subsequently, HepG2 and PLC/PRF/5 cells were co‐cultured with CD8^+^ T cells under varied conditions: control (vehicle treatment), anlotinib treatment, and TFRC knockdown (siTFRC). Treatment with anlotinib led to a higher level of CD8^+^ T‐cell recruitment compared with cells treated with the vehicle control (all *p*‐values < 0.05, Figure 5C, Figure S7D). In both HepG2 and PLC/PRF/5 cells, *TFRC* knockdown resulted in a significant increase in CD8^+^ T‐cell recruitment compared with the control group (all *p*‐values < 0.05, Figure 5C, Figure S7D). These findings revealed an inverse relationship between TFRC expression and CD8^+^ T‐cell recruitment in HCC, where TFRC knockdown leads to enhanced recruitment of CD8^+^ T cells. Furthermore, we observed a reduction in the viability of both HepG2 and PLC/PRF/5 cells in the siTFRC and anlotinib treatment group when they were co‐cultured with CD8^+^ T cells (all *p*‐values < 0.05, Figure 5D, Figure S7D). This indicates that TFRC expression plays a crucial role in regulating the efficacy of CD8^+^ T‐cell infiltration, particularly in the context of anlotinib‐induced immunomodulation.

Since the migration of immune cells appears to be crucial for the increased sensitivity of HCC to anti‐PD‐1 following anlotinib treatment, we next explored the chemokines involved in the infiltration of CD8^+^ T cells. We focused on the chemokines that were differentially expressed (*p* < 0.05 and |log2FC| > 2) in situ in the tumour samples from HCC‐bearing mice. The intersecting differentially expressed cytokines identified were CXCL14 and IL33 (interleukin 33). Notably, CXCL14 exhibited the most significant differences in terms of both the *p*‐value and fold change (Figure 5E).

Recent studies have described a role for CXCL14 in the recruitment of CD8^+^ T cells.^24^ A transcriptomic analysis of TCGA showed that there was a correlation between high CXCL14 expression and increased CD8^+^ T‐cell prevalence, along with elevated immune microenvironment scores (Figure S7E, F). To investigate the chemotactic effects of CXCL14 on CD8^+^ T‐cell mobilization, we performed a transwell assay with four distinct experimental conditions (Figure 5F). This included a control group without CXCL14 (Vehicle) and three experimental groups with 25, 50, and 100 ng/mL CXCL14 in the lower chamber. Following a 48 h incubation, the migration of CD8^+^ T cells was quantified. Our results indicated that there was a dose‐dependent increase in cell migration, with the 50 and 100 ng/mL concentrations demonstrating significant enhancements in CD8^+^ T‐cell recruitment (*p*‐value < 0.05, Figure 5F).

To ascertain if CXCL14 is released by tumour cells, HepG2 and PLC/PRF/5 cells were treated with varying concentrations of anlotinib for 48 h, after which both mRNA and protein levels of CXCL14 were evaluated in the tumour cells and their culture supernatants. The levels of both mRNA and protein for CXCL14 increased significantly in an anlotinib dose‐dependent manner (Figure S8A, B). Additionally, ELISA results demonstrated a significant elevation in CXCL14 levels in the anlotinib‐treated group compared with the control group (*p*‐value < 0.05, Figure S8C). Significantly, the concentration of CXCL14 in the culture medium was notably elevated in the group receiving combination therapy relative to the group treated solely with anti‐PD‐1 monotherapy (all *p*‐values < 0.05, Figure S8C). These results indicate that anlotinib upregulates the secretion of CXCL14 from HCC cells.

To further investigate the role of CXCL14, we assessed CXCL14 mRNA and protein expression in tumour cells, as well as protein concentrations in the culture supernatants, following TFRC silencing in HepG2 and PLC/PRF/5 cells. We observed a significant elevation of CXCL14 expression in tumour cells following TFRC knockdown, a result that aligns with the increased expression observed after anlotinib administration (*p*‐value < 0.05, Figures S9A–C and S10A). Immunohistochemical analyses of mouse tumours with TFRC knockdown revealed that both CXCL14 expression and the CD8^+^ T‐cell counts were significantly increased in the shTFRC group compared with the control group. Likewise, the combination treatment group (shTFRC+anti‐PD‐1) exhibited significantly higher CXCL14 expression and more CD8^+^ T cells compared with the anti‐PD‐1 monotherapy group (*p* < 0.001, Figure 5G). Thus, anlotinib inhibits the expression of TFRC and induces the secretion of CXCL14 by HCC cells, facilitating the infiltration of CD8^+^ T cells into the TME.

### 3.6 TFRC is associated with the response to anti‐PD‐1‐based immunotherapy and the survival of HCC patients

**FIGURE 6:**
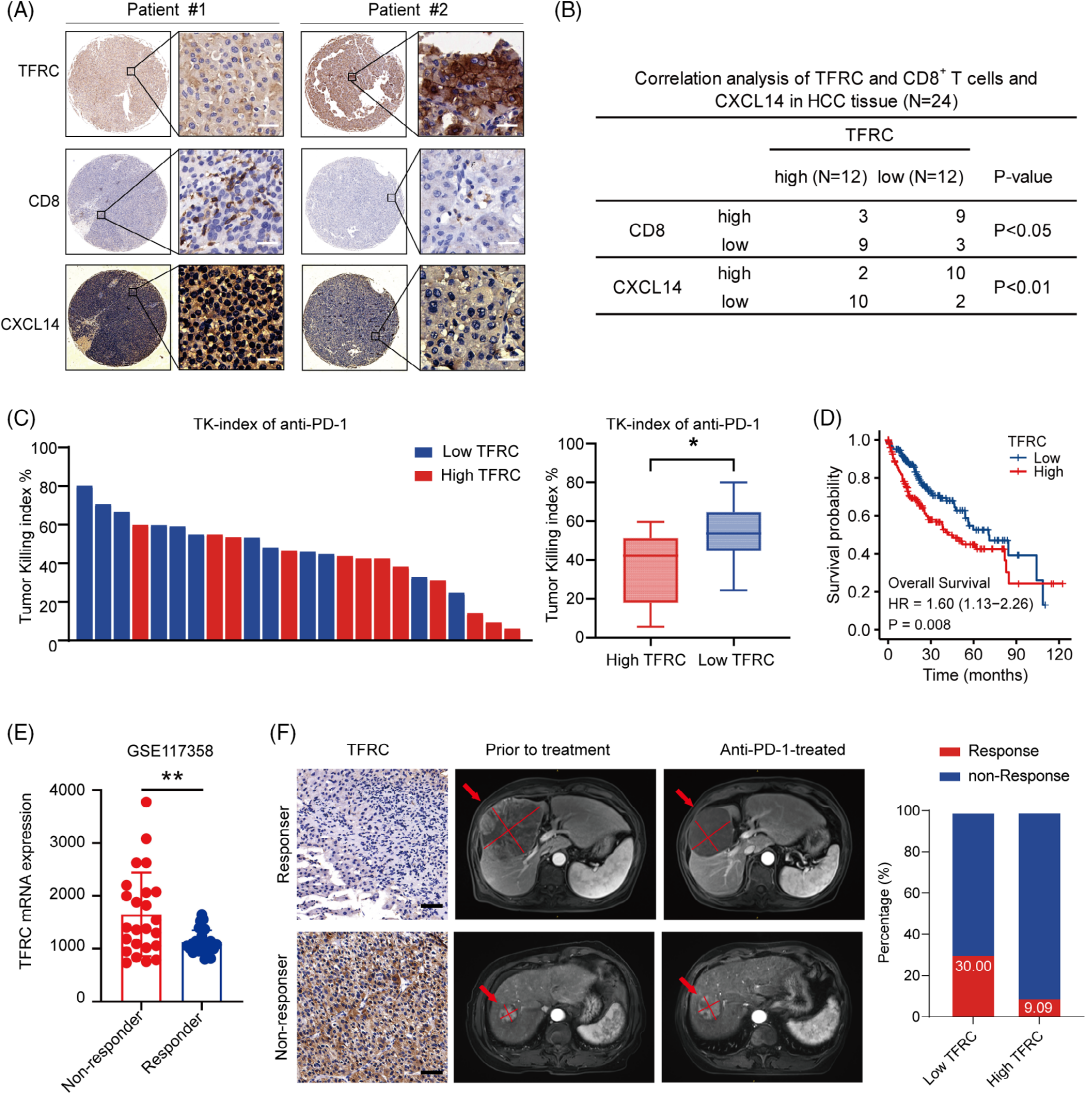
TFRC is associated with the overall survival and response of patients with HCC to anti‐PD‐1‐based immunotherapy. (A) IHC analysis of TFRC protein expression, CXCL14 secretion and CD8^+^ T‐cell infiltration in the primary HCC used to establish the PDOTs. Scale bar: 25 μm. (B) Correlations among the TFRC and CXCL14 protein expression and CD8^+^ T‐cell infiltration in HCC tissues. *p*‐value by Pearson's χ^2^ analysis. (C) Bar graph showing the TKI (tumour‐killing index) of different samples from PDOTs following anti‐PD‐1 treatment. Box plot analysis of the TKI in groups with high and low TFRC expression. (D) Kaplan–Meier curves for the overall patient survival stratified according to the TFRC expression (high/low) in the TCGA database. (E) Dot plot depicting the TFRC mRNA expression levels from the GSE117358 dataset, classified as non‐responders and responders to anti‐PD‐1 therapy (**p < 0.01). (F) IHC analysis of the TFRC expression in primary HCC (left), scale bar: 100 μm. CT images of lesions before and after treatment with anlotinib plus pembrolizumab (right). Data are presented as mean ± SD, *p < 0.05; **p < 0.01; ***p < 0.001; ns, not significant.

We next performed immunohistochemical staining on 24 HCC samples (corresponding to the cohort used for the PDOTs analysis, Table S1). The patients with low TFRC expression showed high expression of CXCL14 and harboured significantly more CD8^+^ T cells in tumour tissues compared with the patients with high TFRC expression (all *p*‐values < 0.05, Figure 6A, B). Compared with the PDOTs from patients with low TFRC expression, those from patients with high TFRC expression exhibited weaker tumour‐killing effects following anti‐PD‐1 antibody treatment (*p* < 0.05, Figure 6C). Notably, for patients exhibiting higher TFRC expression, co‐administration of anlotinib substantially amplified the TK‐index compared with monotherapy with anti‐PD‐1 (*p* < 0.001, Figure S10B). However, in those with low TFRC expression, the increase in the TK‐index following the combined treatment was not statistically significant (*p* = 0.064, Figure S10).

Using the TCGA database, we also found that HCC patients with low expression of TFRC had a significantly better prognosis (overall survival), than those with high TFRC expression (Figure 6D). Remarkably, when we investigated the GSE117358 cohort from the GEO database, the data also indicated that the patients exhibiting low TFRC expression had a more favorable response to anti‐PD‐1 therapy compared with the high expression group (Figure 6E). To validate our findings, we recruited 21 patients with advanced HCC who received anti‐PD‐1‐based treatment and we observed an objective response rate of 19.05% and a disease control rate of 61.90% in this cohort. The patients with low TFRC expression in their resected tumour specimens exhibited a stronger response to anti‐PD‐1‐based treatment (30% vs. 9.09%, *p* < 0.01, Figure 6F). These findings indicate that low TFRC expression is associated with a better prognosis and better response of HCC to anti‐PD‐1‐based therapy.

## 4 DISCUSSION

**FIGURE 7:**
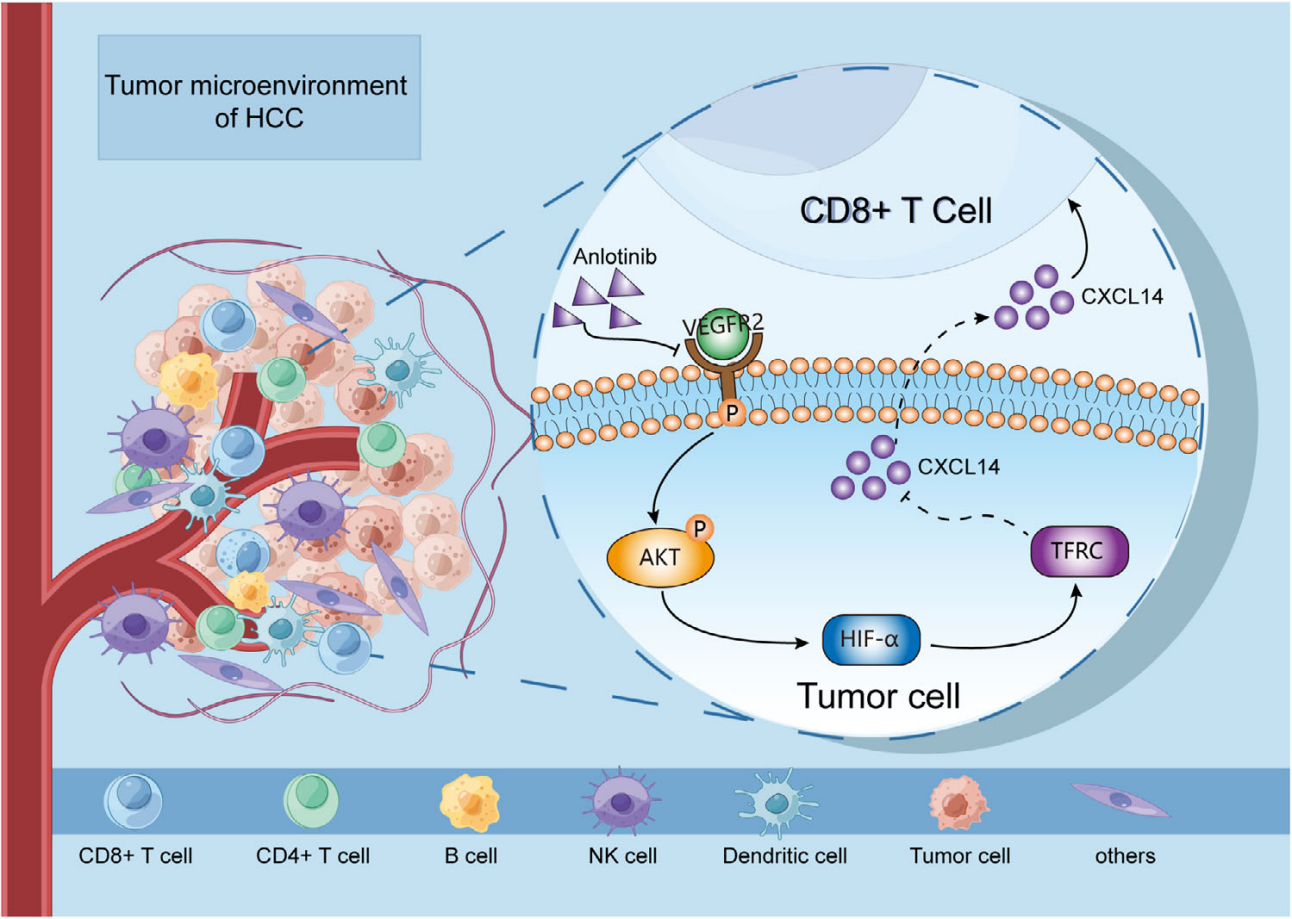
Schematic depiction of the mechanism underlying how anlotinib enhances the tumour response to anti‐PD‐1 immunotherapy.

The introduction of ICI‐based immunotherapy has revolutionized the treatment of cancer patients, including those with advanced HCC.^28^ We herein confirmed that anlotinib acts synergistically with anti‐PD‐1 treatment in HCC. Our findings indicate that anlotinib suppresses TFRC expression through the PI3K/AKT/HIF‐α pathway, which consequently upregulates CXCL14 gene expression, recruiting CD8^+^ T cells into the TME, which improves the efficacy of anti‐PD‐1 treatment (Figure 7). Moreover, the association of low TFRC expression in tumour specimens with enhanced responsiveness to anti‐PD‐1‐based immunotherapy suggests that TFRC levels could serve as a biomarker for predicting the treatment efficacy of anti‐PD‐1‐based approaches in HCC patients.

Using combinations of targeted therapies may induce immunogenic cell death in tumour cells, fostering tumour antigen presentation, and resulting in improved tumour eradication.^29^, ^30^ Although these effects of anlotinib were described previously,^31^ our present research uncovered the mechanism by which anlotinib improves the efficacy of anti‐PD1 immunotherapy for HCC. Anlotinib specifically suppresses the expression of TFRC in HCC cells via the PI3K/AKT/HIF‐α pathway and consequently leads to the upregulation of CXCL14 gene expression within these cells. CXCL14 may contribute to the recruitment of CD8^+^ T cells under certain conditions, although further studies are needed to establish a definitive role in HCC. This ultimately results in reshaping the TME and enhancing anti‐tumour immune responses, thereby enhancing the effectiveness of anti‐PD‐1 therapy.

We discovered that anlotinib triggers a significant influx of both CD4^+^ and CD8^+^ T cells to tumour locations, which is mediated by the release of inflammatory cytokines and chemokines. Depletion studies utilizing anti‐CD4 and anti‐CD8 neutralizing antibodies or isotype control IgG revealed that the effects were predominantly attributed to the action of anti‐CD8^+^ T cells. This evidence highlights the pivotal role that CD8^+^ T cells play in driving tumour regression in response to this combination therapy. Furthermore, the majority of the CD8^+^ T cells expressed a PD‐1^high^ phenotype. Since this phenotype indicates a functionally exhausted status, adding anti‐PD‐1 antibodies would restore the cytotoxic function of these cells by relieving the immune‐inhibitory signals.

In the current study, we found that TFRC is an integral downstream effector in the PI3K/AKT/HIF‐α signaling pathway. Specifically, TFRC^Low^ significantly facilitates the infiltration of immune cells into the TME. Although such studies are beyond the scope of the present manuscript, we speculate that TFRC might negatively influence CXCL14 via enhancer of zeste homolog 2 (EZH2). Previous research reported a positive correlation between the expression levels of TFRC and EZH2,^32^ and there was an increase in CXCL14 expression when EZH2 was conditionally knocked down in mice.^33^ CXCL14, a member of the CXC chemokine family, exhibits a significant chemotactic impact on CD8^+^ T cells and monocytes.^34^, ^35^, ^36^ It serves as a vital link between the tumour and the immune system, attracting CD8^+^ T cells to the tumour location and enhancing their tumour‐fighting capabilities. Further investigations are required to elucidate the regulatory mechanisms behind CXCL14 expression that enhance CD8^+^ T‐cell recruitment, along with the resulting anti‐tumour actions of these cells.

We used a novel microfluidic PDOTs model to evaluate the efficacy of immunotherapy drugs in this study because this model simulates the tumour immune microenvironment, which is lacking in traditional in vitro and many in vivo tumour models.^37^ Its wide application in the liver and other cancer research will enhance the precision of immune‐oncology and promote the development of more effective therapies, including better combinations for anti‐PD‐1‐based treatments.

Several limitations to our study should be acknowledged. We demonstrated the efficacy of merging anlotinib with anti‐PD‐1 treatment in preclinical models and highlighted the significance of the identified target molecule in a small cohort of HCC patients. However, it is crucial to acknowledge the necessity for further validation through extensive, prospective, randomized controlled trials. Whether the synergistic amplification of the anti‐PD‐1 antibody's effects can be replicated with other TKIs requires additional investigation. Furthermore, the potential toxicity and off‐target effects of the combination therapies should also be assessed. Further investigations on the specific mechanisms underlying how TFRC regulates CXCL14 are also necessary.

In conclusion, our study suggests that combining anlotinib and anti‐PD‐1 immunotherapy represents a promising therapeutic strategy for advanced HCC patients, providing a theoretical basis and preclinical data to support future clinical trials. Mechanistically, anlotinib suppresses the expression of TFRC by inhibiting the VEGFR2‐AKT‐HIF axis, which in turn enhances the secretion of CXCL14 and recruits tumour‐infiltrating CD8^+^ T cells, particularly PD‐1^+^CD8^+^ T cells, thereby potentiating the anti‐tumour efficacy of anti‐PD‐1 treatment. Furthermore, TFRC might be a potential biomarker for predicting the responses of tumours to anti‐PD‐1‐based therapies and the overall survival of HCC patients.

## AUTHOR CONTRIBUTIONS

Xin‐Rong Yang, Zhong Chen, and Fei Song designed the study. Bo Hu and Xiao‐Liang Liang interpreted the data. Fei Song and Xin‐Rong Yang wrote the manuscript. Cheng‐Gui Wang, Peng‐Xiang Wang, Tian‐Lun Wang, and Peng‐Ju Tang conducted most of the experiments. Hai‐Xiang Sun, Wei Guo, Jian Zhou and Jian‐Wen Cheng helped with the design of experiments, coordinated the study, and contributed key reagents. Jia Fan, Zhong Chen and Xin‐Rong Yang provided study supervision and were responsible for the final approval of the manuscript.

## CONFLICT OF INTEREST STATEMENT

The authors declare no conflict of interest.

## ETHICS STATEMENT AND CONSENT TO PARTICIPATE

This study was performed in accordance with the 1975 Declaration of Helsinki. Approval for the use of human subjects was obtained from the Research Ethical Committee of Zhongshan Hospital, and informed consent was obtained from each individual enrolled in this study.

## Supporting information

Supporting Information

## Data Availability

All data generated or analyzed during this study are included in this published article (and the Supporting Information).

## References

[1] He X , Xu C . Immune checkpoint signaling and cancer immunotherapy. Cell Res. 2020;30:660‐669. 32467592 10.1038/s41422-020-0343-4 PMC7395714

[2] Minton K . Immune checkpoint blockade breaches the mucosal firewall to induce gut microbiota translocation. Nat Rev Immunol. 2023;23:269. 36918665 10.1038/s41577-023-00865-x

[3] Llovet JM , Kelley RK , Villanueva A , et al. Hepatocellular carcinoma. Nat Rev Dis Primers. 2021;7:6. 33479224 10.1038/s41572-020-00240-3 PMC13537433

[4] Meric‐Bernstam F , Larkin J , Tabernero J , Bonini C . Enhancing anti‐tumour efficacy with immunotherapy combinations. Lancet. 2021;397:1010‐1022. 33285141 10.1016/S0140-6736(20)32598-8

[5] Yau T , Kang YK , Kim TY , et al. Efficacy and safety of nivolumab plus ipilimumab in patients with advanced hepatocellular carcinoma previously treated with sorafenib: the CheckMate 040 randomized clinical trial. JAMA Oncol. 2020;6:e204564. 33001135 10.1001/jamaoncol.2020.4564 PMC7530824

[6] Zhu XD , Sun HC . Emerging agents and regimens for hepatocellular carcinoma. J Hematol Oncol. 2019;12:110. 31655607 10.1186/s13045-019-0794-6 PMC6815423

[7] Finn RS , Ryoo BY , Merle P , et al. Pembrolizumab as second‐line therapy in patients with advanced hepatocellular carcinoma in KEYNOTE‐240: a randomized, double‐blind, phase III trial. J Clin Oncol. 2020;38:193‐202. 31790344 10.1200/JCO.19.01307

[8] Chu Y , Dai E , Li Y , et al. Pan‐cancer T cell atlas links a cellular stress response state to immunotherapy resistance. Nat Med. 2023;29:1550‐1562. 37248301 10.1038/s41591-023-02371-y PMC11421770

[9] Morad G , Helmink BA , Sharma P , Wargo JA . Hallmarks of response, resistance, and toxicity to immune checkpoint blockade. Cell. 2021;184:5309‐5337. 34624224 10.1016/j.cell.2021.09.020 PMC8767569

[10] Yi C , Chen L , Lin Z , et al. Lenvatinib targets FGF receptor 4 to enhance antitumor immune response of anti‐programmed cell death‐1 in HCC. Hepatology. 2021;74:2544‐2560. 34036623 10.1002/hep.31921

[11] Shen G , Zheng F , Ren D , et al. Anlotinib: a novel multi‐targeting tyrosine kinase inhibitor in clinical development. J Hematol Oncol. 2018;11:120. 30231931 10.1186/s13045-018-0664-7 PMC6146601

[12] Su Y , Luo B , Lu Y , et al. Anlotinib induces a T cell‐inflamed tumor microenvironment by facilitating vessel normalization and enhances the efficacy of pd‐1 checkpoint blockade in neuroblastoma. Clin Cancer Res. 2022;30:793‐809. 10.1158/1078-0432.CCR-21-2241 PMC9377760 34844980

[13] Xu Q , Wang J , Sun Y , et al. Efficacy and safety of sintilimab plus anlotinib for PD‐L1‐positive recurrent or metastatic cervical cancer: a multicenter, single‐arm, prospective phase II trial. J Clin Oncol. 2022;40:1795‐1805. 35192397 10.1200/JCO.21.02091 PMC9148684

[14] Chu T , Zhong R , Zhong H , et al. Phase 1b study of sintilimab plus anlotinib as first‐line therapy in patients with advanced NSCLC. J Thorac Oncol. 2021;16:643‐652. 33524601 10.1016/j.jtho.2020.11.026

[15] Yang XR , Xu Y , Yu B , et al. High expression levels of putative hepatic stem/progenitor cell biomarkers related to tumour angiogenesis and poor prognosis of hepatocellular carcinoma. Gut. 2010;59:953‐962. 20442200 10.1136/gut.2008.176271

[16] Llovet JM , Lencioni R . mRECIST for HCC: performance and novel refinements. J Hepatol. 2020;72:288‐306. 31954493 10.1016/j.jhep.2019.09.026 PMC12452114

[17] Cañadas I , Thummalapalli R , Kim JW , et al. Tumor innate immunity primed by specific interferon‐stimulated endogenous retroviruses. Nat Med. 2018;24:1143‐1150. 30038220 10.1038/s41591-018-0116-5 PMC6082722

[18] Jenkins RW , Aref AR , Lizotte PH , et al. Ex vivo profiling of PD‐1 blockade using organotypic tumor spheroids. Cancer Discov. 2018;8:196‐215. 29101162 10.1158/2159-8290.CD-17-0833 PMC5809290

[19] Sun HX , Xu Y , Yang XR , et al. Hypoxia inducible factor 2 alpha inhibits hepatocellular carcinoma growth through the transcription factor dimerization partner 3/E2F transcription factor 1‐dependent apoptotic pathway. Hepatology. 2013;57:1088‐1097. 23212661 10.1002/hep.26188 PMC3594482

[20] Chou TC . Theoretical basis, experimental design, and computerized simulation of synergism and antagonism in drug combination studies. Pharmacol Rev. 2006;58:621‐681. 16968952 10.1124/pr.58.3.10

[21] Chen X , Li W , Wu X , et al. Safety and efficacy of sintilimab and anlotinib as first line treatment for advanced hepatocellular carcinoma (KEEP‐G04): a single‐arm phase 2 study. Front Oncol. 2022;12:909035. 35712486 10.3389/fonc.2022.909035 PMC9197581

[22] Hu B , Xu Y , Li YC , et al. CD13 promotes hepatocellular carcinogenesis and sorafenib resistance by activating HDAC5‐LSD1‐NF‐kappaB oncogenic signaling. Clin Transl Med. 2020;10:e233. 33377659 10.1002/ctm2.233 PMC7708822

[23] Gao J , Shi LZ , Zhao H , et al. Loss of IFN‐γ pathway genes in tumor cells as a mechanism of resistance to anti‐CTLA‐4 therapy. Cell. 2016;167:397‐404. 27667683 10.1016/j.cell.2016.08.069 PMC5088716

[24] Coelho MA , Cooper S , Strauss ME , et al. Base editing screens map mutations affecting interferon‐gamma signaling in cancer. Cancer Cell. 2023;41:288‐303. 36669486 10.1016/j.ccell.2022.12.009 PMC9942875

[25] Qi D , Lu M , Xu P , et al. Transcription factor ETV4 promotes the development of hepatocellular carcinoma by driving hepatic TNF‐alpha signaling. Cancer Commun (Lond). 2023;43:1354‐1372. 37670477 10.1002/cac2.12482 PMC10693303

[26] Kim H , Villareal LB , Liu Z , et al. Transferrin receptor‐mediated iron uptake promotes colon tumorigenesis. Adv Sci (Weinh). 2023;10:e2207693. 36703617 10.1002/advs.202207693 PMC10074045

[27] Xiao X , Moschetta GA , Xu Y , et al. Regulation of iron homeostasis by hepatocyte TfR1 requires HFE and contributes to hepcidin suppression in beta‐thalassemia. Blood. 2023;141:422‐432. 36322932 10.1182/blood.2022017811 PMC9936306

[28] Cheng AL , Hsu C , Chan SL , Choo SP , Kudo M . Challenges of combination therapy with immune checkpoint inhibitors for hepatocellular carcinoma. J Hepatol. 2020;72:307‐319. 31954494 10.1016/j.jhep.2019.09.025

[29] Clucas J , Meier P . Roles of RIPK1 as a stress sentinel coordinating cell survival and immunogenic cell death. Nat Rev Mol Cell Biol. 2023;24:835‐852. 37568036 10.1038/s41580-023-00623-w

[30] Galluzzi L , Vitale I , Warren S , et al. Consensus guidelines for the definition, detection and interpretation of immunogenic cell death. J Immunother Cancer. 2020;8:e000337. 32209603 10.1136/jitc-2019-000337 PMC7064135

[31] Song F , Hu B , Cheng JW , et al. Anlotinib suppresses tumor progression via blocking the VEGFR2/PI3K/AKT cascade in intrahepatic cholangiocarcinoma. Cell Death Dis. 2020;11:573. 32709873 10.1038/s41419-020-02749-7 PMC7381674

[32] Hou S , Clement RL , Diallo A , et al. FoxP3 and Ezh2 regulate Tfr cell suppressive function and transcriptional program. J Exp Med. 2019;216:605‐620. 30705058 10.1084/jem.20181134 PMC6400538

[33] Wang J , Yu X , Gong W , et al. EZH2 noncanonically binds cMyc and p300 through a cryptic transactivation domain to mediate gene activation and promote oncogenesis. Nat Cell Biol. 2022;24:384‐399. 35210568 10.1038/s41556-022-00850-x PMC9710513

[34] Westrich JA , Vermeer DW , Silva A , et al. CXCL14 suppresses human papillomavirus‐associated head and neck cancer through antigen‐specific CD8(+) T‐cell responses by upregulating MHC‐I expression. Oncogene. 2019;38:7166‐7180. 31417179 10.1038/s41388-019-0911-6 PMC6856418

[35] Kumar A , Mohamed E , Tong S , et al. CXCL14 promotes a robust brain tumor‐associated immune response in glioma. Clin Cancer Res. 2022;28:2898‐2910. 35511927 10.1158/1078-0432.CCR-21-2830 PMC9250623

[36] Dolinska M , Cai H , Mansson A , et al. Characterization of the bone marrow niche in patients with chronic myeloid leukemia identifies CXCL14 as a new therapeutic option. Blood. 2023;142:73‐89. 37018663 10.1182/blood.2022016896 PMC10651879

[37] Hu B , Li H , Guo W , et al. Establishment of a hepatocellular carcinoma patient‐derived xenograft platform and its application in biomarker identification. Int J Cancer. 2020;146:1606‐1617. 31310010 10.1002/ijc.32564

