# Supplementary material for: Anlotinib potentiates anti‐PD1 immunotherapy via transferrin receptor‐dependent CD8+ T‐cell infiltration in hepatocellular carcinoma
Source: Clin Transl Med. 2024 Aug 2;14(8):e1738. doi: 10.1002/ctm2.1738 (PMC11296886; doi:10.1002/ctm2.1738)

**Anlotinib potentiates anti-PD1 immunotherapy via transferrin receptor-dependent CD8+ T-cell infiltration in hepatocellular carcinoma**

**Supplementary Methods**

**HCC cell lines and reagents**

The Liver Cancer Institute of Zhongshan Hospital provided the PLC/PRF/5, HepG2, Hepa1-6, and H22 cells. All human and mouse liver cancer cells were cultured in DMEM or MEM with 10% FBS and 1% penicillin-streptomycin (Beyotime, C0222) at 37 °C with 5% CO_2_. The anti-mouse PD-1 monoclonal antibody (InVivoMAb anti-mouse PD-1, CD279) was purchased from BioXCell. Anlotinib was provided by Zhengda Tianqing Pharmaceutical Group, while sintilimab was supplied by Innovent Biologics (Suzhou, China) Co., Ltd. Dimethyl sulfoxide (DMSO) was used to dissolve the anlotinib, and sintilimab was dissolved in normal saline. Working solutions were prepared by diluting the stock solutions.

**Spheroid preparation and microfluidic culture**

To avoid differences in immune cell content caused by heterogeneity in sample collection areas, samples were collected from multiple different regions of the tumor. The core and peripheral tumor areas were mixed to ensure a representative distribution of immune cells within the tumor. Experiments were performed as described previously [1]. Briefly, fresh tumor specimens from human patients were received in media (DMEM) on ice and minced in 6 cm dishes (on ice) in a sterile field. The S2 fractions (40–100 µm) were used for ex vivo culture. An aliquot of the S2 fraction was pelleted and resuspended in type I rat tail collagen, and the spheroid-collagen mixture was then injected into the center gel region of the 3D microfluidic culture device. After 30 minutes at 37°C, collagen hydrogels containing PDOTs were hydrated with media containing the indicated treatments. PDOTs were grown using Advanced DMEM/F12 (Gibco, 12634-010), 10% Fetal Bovine Serum (Gibco, 10270-106), IL-2 (500U/ml, Peprotech, 200-02-100), and ImmunoCult™ Human CD3/CD28 T-cell Activator (STEMCELL, 10971) for 6 days. PDOTs were treated with anlotinib (10 μM), sintilimab (500 ug/ml), or a combination.

**Live/dead staining and immunofluorescence**

Dual labeling was performed by loading a microfluidic device with Nexcelom ViaStain™ AO/PI Staining Solution [2]. The PDOTs were fixed with 4% paraformaldehyde, saponin, and then blocked with QuickBlock™ Blocking Buffer for Immunostaining. Cells were incubated overnight with the indicated directly conjugated antibodies (CD45, Abcam, ab200315, Alexa Fluor® 488; CD8, Bioss, bs-0648R, Alexa Fluor® 488) [3,4]. The nuclei were stained with DAPI. Images were visualized using a confocal microscope.

**Transcriptome sequencing (RNA-Seq) and analysis**

RNA integrity was assessed using the RNA Nano 6000 Assay Kit and the Bioanalyzer 2100 system (Agilent Technologies, CA, USA). mRNA molecules were purified from total RNA using oligo (dT)-attached magnetic beads and fragmented using fragmentation reagent. First-strand cDNA was generated using random hexamer-primed reverse transcription, followed by second-strand cDNA synthesis. The PCR products were purified, and the library quality was assessed on the Agilent Bioanalyzer 2100 system. The index-coded samples were clustering on a cBot Cluster Generation System using TruSeq PE Cluster Kit v3-cBot-HS (Illumina, CA, USA), the library preparations were sequenced on an Illumina Novaseq platform, and 150 bp paired-end reads were generated. The differential expression analysis of two groups was performed using the DESeq2 R package (1.20.0). A gene was considered significantly differentially expressed when it had a fold change > 2 and adjusted P-value < 0.05, as calculated by DESeq2.

The Gene Ontology (GO) and Kyoto Encyclopedia of Genes and Genomes (KEGG) analyses were performed by cluster profile, where the differentially expressed genes identified as described above were supplied as the input for genes of interest and functional profiles were compared by the compareCluster function. GSEA was performed on a list of genes ranked according to limma PI-values, using the GSEAPreRanked function with enrichment statistic classic and 1000 permutations [5]. For specific enrichment of super-enhancer-associated genes found in a custom gene set, the genes identified above were first extracted and merged within the GO BP category of the MSigDB database to obtain the enrichment ranking.

**Time-of-flight mass cytometry (CyTOF) and analysis**

Mouse tumor samples were mechanically dissociated and enzymatically digested using Liberase DL in Hanks balanced salt solution for 30 min at 37°C. The solution was passed through a 70-mm cell strainer to obtain a single-cell suspension. Tumor-infiltrating immune cells were separated by 30% and 70% gradient centrifugation. Immune cells were washed once with 1xPBS and then stained with 100 μL of 250 nM cisplatin for 5 min on ice to exclude dead cells, and then were incubated in Fc receptor blocking solution before being stained with a surface antibodies cocktail for 30 min on ice. Cells were washed twice with FACS buffer (1×PBS+0.5%BSA) overnight and fixed in 200 μL of intercalation solution (Maxpar Fix and Perm Buffer containing 250 nM 191/193Ir). After fixation, cells were washed once with FACS buffer and then Perm buffer and stained with an intracellular antibodies cocktail for 30 min on ice. Cells were then washed and resuspended with deionized water, added to 20% EQ beads, and data were acquired using a mass cytometer.

The raw data for each sample were analyzed using a doublet-filtering scheme with unique mass-tagged barcodes. The bead normalization method normalized each .fcs file generated from different batches. Data were manually gated using the FlowJo software to exclude debris, dead cells, and doublets, leaving live, single immune cells. The PhenoGraph clustering algorithm was applied to all cells to partition them into distinct phenotypes based on their marker expression levels. The cell type of each cluster was annotated according to its marker expression pattern on a heatmap of cluster vs. marker. The dimensionality reduction algorithm (t-SNE) was used to visualize the data in two dimensions and show the distribution of each cluster and marker expression, as well as the differences among each group or among different sample types. Statistical analyses of the frequency of annotated cell populations were performed using t-tests [6].

**Bioinformatic analysis**

The assessment of tumor-infiltrating immune cells was performed using the CIBERSORT database (https://cibersort.stanford.edu/) and ImmuCellAI database (http://bioinfo.life.hust.edu.cn/ImmuCellAI/). A leukocyte gene signature matrix containing 547 genes was used to characterize immune cell subtypes from 22 human species based on the CIBERSORT database. Immune Cell Abundance Identifier, a gene set signature-based method, was used to precisely estimate the abundance of 24 immune cell types, including 18 T-cell subsets, from gene expression data. The RNA-seq data from Liver Hepatocellular Carcinoma (LIHC) patients were obtained from the Cbioportal (http://www.cbioportal.org/). The CIBERSORT algorithm and ssGSEA algorithm were used to process the RNA-seq data, and the proportions of different immune cell populations were acquired using the ‘Cell Fraction Analysis’ module [7,8].

**Quantitative real-time PCR**

Total RNA was isolated from cultured human cancer cells or mouse tumors using a FastPure Cell/Tissue Total RNA Isolation Kit V2 according to the standard protocol. RNA was reverse-transcribed using the Hifair^®^ II 1st Strand cDNA Synthesis Kit according to the manufacturer’s instructions. Quantitative PCR was performed using Hieff UNICON^®^ qPCR SYBR Green Master Mix. All mRNA expression levels were normalized to GAPDH and calculated using the 2−^△△^CT method [9]. The specific primer sequences used in the study can be found in **Supplementary Table S3**.

**Western blotting**

Cells were lysed in cold RIPA buffer in the presence of 1×protease inhibitor cocktail and 1×PhosStop (Roche, Isere, France) after two washes in phosphate-buffered saline (PBS). After centrifugation, cell supernatants were collected. The protein concentrations in the lysates were measured by the Pierce bicinchoninic acid (BCA) protein assay kit. Subsequently, SDS loading buffer (5×) was added to each sample, and samples were heated at 100°C for 10 min. Equal amounts of protein were resolved by SDS–PAGE and transferred to a PVDF film transfer membrane (Millipore, ISEQ00010). The membrane was blocked in Quick-Block™ Blocking Buffer for Western Blot at room temperature for 0.5 hours and incubated with appropriate antibodies at 4°C overnight. Antibodies were diluted in Quick-Block™ Primary Antibody Dilution Buffer for Western Blot. On the next day, the membrane was washed with TBST (TBS with 0.1% Tween) three times and incubated with appropriate secondary antibodies at room temperature for 3 hours [10]. Membranes were imaged using the LI-COR Odyssey® Imaging System. Information pertaining to the specific antibodies utilized in this study is included in **Supplementary Table S4**.

**Cell transfection**

Gene expression knockdown (KD) was successfully achieved utilizing a short hairpin RNA (shRNA)-mediated silencing approach [11]. The target was the transferrin receptor (TFRC) gene in murine hepatocellular carcinoma (HCC) cell lines. To achieve stably silenced cell lines, shRNA lentiviral vectors specifically designed for TFRC were used: TFRC-1 sense, 5’-CCGGCGTTGAATTGAACCTGGACTCTCGAGTAGTCCAGGTTCAATTCAACGTTTTTG-3’, TFRC-2 sense, 5’-CCGGCCAGACCGTTATGTTGTAGTACTCGAGTACTACAACATAACGGTCTGGTTTTTG-3’ and TFRC-3 sense, 5’-CCGGCGTATTATGAAAGTGGAGTATCTCGAGATACTCCACTTTCATAATACGTTTTTG-3’.

For small interfering RNA (siRNA)-based knockdown, HepG2 and PLC cells were cultured at a density of 4×106 cells/ml in a 24-well plate for 12 hours prior to transfection. The transfection solution was prepared by diluting 2 μl of Lipofectamine 2000 and 120 pmol of siRNA each with 25 μl of Opti-MEM. Subsequently, the diluted Lipofectamine 2000 was combined with the diluted siRNA, and the mixture was allowed to incubate for 20 minutes. Non-targeting siRNA was used as a negative control for these experiments. The siRNA sequences for the TFRC gene were as follows: si-1 sense, 5‘-GGAUCUAUAGUGAUUGUCATT-3‘, antisense, 3‘-UGACAAUCACUAUAGAUCCTT-5’; si-2 sense, 5’-GGUCAUCAGGAUUGCCUAATT-3’, antisense, 3’-UUAGGCAAUCCUGAUGACCTT-5’; si-3 sense, 5’-GCCAGCUUUACUGGAGAACUUTT-3’, antisense, 3’- AAGUUCUCCAGUAAAGCUGGCTT-5’ and si-NC sense, 5’-UUCUCCGAACGUGUCACGUDTDT-3’, antisense, 3’-ACGUGACACGUUCGGAGAADTDT-5’. The RNA duplexes were synthesized by Genomeditech (Shanghai, China). Following transfection, a period of 48 hours was allowed before the mRNA expression level of TFRC was measured using qRT-PCR.

**Immunohistochemistry**

Slides with formalin-fixed and paraffin-embedded tissue sections were deparaffinized using Xylene and serially hydrated by incubating them in decreasing percentages of alcohol (100-50%). Antigen retrieval was achieved by placing the sections in antigen retrieval solution and steam cooking the samples for 1 h. Endogenous peroxidase was depleted with 0.3% hydrogen peroxide. Subsequently, the slides were washed with PBS + 0.1% Tween 20, then blocked with serum. After blocking, slides were incubated overnight with primary antibodies against CD8, CD4, PD-1, CD19, and TFRC at 4°C. The next day, the slides were washed with PBS and incubated in biotinylated goat anti-rabbit secondary antibody for 30 min. Finally, the color was developed by incubating the slides in DAB for 5-10 min [12].

**CD8+ T-cell-mediated tumor cytotoxicity assay**

Human CD8^+^ T-cells were isolated from peripheral blood mononuclear cells using a MojoSort™ Human CD8 T-cell Isolation Kit following the manufacturer’s protocols. Purified CD8^+^ T-cells were cultured in ImmunoCult™ XF T-cell Expansion medium, ImmunoCult Human CD3/CD28 T-cell activator (25 µl/ml), and interleukin-2 (100 U/mL) for 3 days. Cancer cells were seeded in 24-well plates overnight, exposed to the appropriate treatments, and co-cultured with activated CD8^+^ T-cells (1:1) for 48 h. CD8+ T-cells and cell debris were removed by washing the cells with PBS, which was followed by crystal violet staining [13-16].

**Enzyme-Linked Immunosorbent Assay (ELISA)**

Cell culture media was collected from PDOTs culture after 2 days and transferred to 1.5 ml EP tubes. After collection, tubes containing conditioned media were stored at −80°C. Aliquots (100 μl) of experimental samples or quality control products were added to the corresponding well for cytokine analysis. The reaction wells were sealed with plywood paper and incubated for 2 h at room temperature. A CXCL14 antibody was added to each microwell, then the plates were incubated at room temperature for 2 h. Then SAA-HRP, chromo-solution, and stop buffer were added to each microwell, and the plates were incubated for 20 min at room temperature [17]. A curve was generated by plotting the standard concentration as a logarithm with the corresponding OD values, and the best-fit line was determined by regression analysis.

**CD8+ T-cell migration assay**

To study in vitro CD8^+^ T-cell transmigration, CD8^+^ lymphocytes were magnetically enriched from normal donor PBMC using a MojoSort™ Human CD8 T-cell Isolation Kit (BioLegend, 480011). Cells were cultured in ImmunoCult™ XF T-cell Expansion Medium (STEMCELL Technologies; Catalog #10981) with ImmunoCult Human CD3/CD28 T-cell activator (25 µl/ml; STEMCELL Technologies) and interleukin-2 (100 U/mL, Peprotech) for 3 days. Then, CD8^+^ T-cells (2.5 × 105) were loaded into the top chamber of Transwell inserts (5.0 μm pore size, Costar). The bottom well was filled with media with or without recombinant human CXCL14 (Peprotech, 300-50). Cells were incubated at 37°C for 48 h, after which the contents of the lower chamber were collected, and the percentage of CD8^+^ T-cells in the bottom chamber was determined using the Countess Cell Counter System (Thermo Fisher Scientific) [17].

**Statistical methods**

Statistical analyses were performed using SPSS version 23.0 and Prism 8 software (GraphPad) or the R programming language (v4.0.3) supported by the R Foundation for Statistical Computing. Quantitative variables were presented as mean ± standard deviation. Where the assumptions of normality and equal variance were met, differences between groups were analyzed using Student’s t-test or one-way ANOVA followed by Tukey’s honestly significant difference post hoc test for multiple comparisons. In cases where these assumptions were not satisfied, nonparametric tests (Wilcoxon rank-sum test) were applied. The Kaplan–Meier method was used to plot the OS curves and data were compared using a log-rank test. The Pearson correlation coefficient was calculated to analyze the correlation between gene mRNA expression and the proportion of immune cells. Differences with P values less than 0.05 were considered statistically significant [18].

**Reference**

1. Jenkins RW, Aref AR, Lizotte PH, Ivanova E, Stinson S, Zhou CW, Bowden M, et al. Ex Vivo Profiling of PD-1 Blockade Using Organotypic Tumor Spheroids. Cancer Discov 2018;8:196-215.

2. Deng J, Wang ES, Jenkins RW, Li S, Dries R, Yates K, Chhabra S, et al. CDK4/6 Inhibition Augments Antitumor Immunity by Enhancing T-cell Activation. Cancer Discov 2018;8:216-233.

3. Zhang Y, McMullen A, Pontani LL, He X, Sha R, Seeman NC, Brujic J, et al. Sequential self-assembly of DNA functionalized droplets. Nat Commun 2017;8:21.

4. Fischer A, Zundler S, Atreya R, Rath T, Voskens C, Hirschmann S, Lopez-Posadas R, et al. Differential effects of alpha4beta7 and GPR15 on homing of effector and regulatory T cells from patients with UC to the inflamed gut in vivo. Gut 2016;65:1642-1664.

5. Fassl A, Brain C, Abu-Remaileh M, Stukan I, Butter D, Stepien P, Feit AS, et al. Increased lysosomal biomass is responsible for the resistance of triple-negative breast cancers to CDK4/6 inhibition. Sci Adv 2020;6:eabb2210.

6. Ogrodnik M, Zhu Y, Langhi LGP, Tchkonia T, Kruger P, Fielder E, Victorelli S, et al. Obesity-Induced Cellular Senescence Drives Anxiety and Impairs Neurogenesis. Cell Metab 2019;29:1061-1077 e1068.

7. Liu D, Schilling B, Liu D, Sucker A, Livingstone E, Jerby-Arnon L, Zimmer L, et al. Integrative molecular and clinical modeling of clinical outcomes to PD1 blockade in patients with metastatic melanoma. Nat Med 2019;25:1916-1927.

8. Tang M, Xie Q, Gimple RC, Zhong Z, Tam T, Tian J, Kidwell RL, et al. Three-dimensional bioprinted glioblastoma microenvironments model cellular dependencies and immune interactions. Cell Res 2020;30:833-853.

9. Ma XL, Shen MN, Hu B, Wang BL, Yang WJ, Lv LH, Wang H, et al. CD73 promotes hepatocellular carcinoma progression and metastasis via activating PI3K/AKT signaling by inducing Rap1-mediated membrane localization of P110beta and predicts poor prognosis. J Hematol Oncol 2019;12:37.

10. Hu B, Xu Y, Li YC, Huang JF, Cheng JW, Guo W, Yin Y, et al. CD13 promotes hepatocellular carcinogenesis and sorafenib resistance by activating HDAC5-LSD1-NF-kappaB oncogenic signaling. Clin Transl Med 2020;10:e233.

11. Song F, Chen FY, Wu SY, Hu B, Liang XL, Yang HQ, Cheng JW, et al. Mucin 1 promotes tumor progression through activating WNT/beta-catenin signaling pathway in intrahepatic cholangiocarcinoma. J Cancer 2021;12:6937-6947.

12. Yang XR, Xu Y, Yu B, Zhou J, Qiu SJ, Shi GM, Zhang BH, et al. High expression levels of putative hepatic stem/progenitor cell biomarkers related to tumour angiogenesis and poor prognosis of hepatocellular carcinoma. Gut 2010;59:953-962.

13. Hartmann FJ, Mrdjen D, McCaffrey E, Glass DR, Greenwald NF, Bharadwaj A, Khair Z, et al. Single-cell metabolic profiling of human cytotoxic T cells. Nat Biotechnol 2021;39:186-197.

14. Li H, Kuang X, Liang L, Ye Y, Zhang Y, Li J, Ma F, et al. The Beneficial Role of Sunitinib in Tumor Immune Surveillance by Regulating Tumor PD-L1. Adv Sci (Weinh) 2021;8:2001596.

15. Nelson N, Lopez-Pelaez M, Palazon A, Poon E, De La Roche M, Barry S, Valge-Archer V, et al. A cell-engineered system to assess tumor cell sensitivity to CD8(+) T cell-mediated cytotoxicity. Oncoimmunology 2019;8:1599635.

16. Liu H, Kuang X, Zhang Y, Ye Y, Li J, Liang L, Xie Z, et al. ADORA1 Inhibition Promotes Tumor Immune Evasion by Regulating the ATF3-PD-L1 Axis. Cancer Cell 2020;37:324-339 e328.

17. Kumar A, Mohamed E, Tong S, Chen K, Mukherjee J, Lim Y, Wong CM, et al. CXCL14 Promotes a Robust Brain Tumor-Associated Immune Response in Glioma. Clin Cancer Res 2022;28:2898-2910.

18. Sun YF, Wu L, Liu SP, Jiang MM, Hu B, Zhou KQ, Guo W, et al. Dissecting spatial heterogeneity and the immune-evasion mechanism of CTCs by single-cell RNA-seq in hepatocellular carcinoma. Nat Commun 2021;12:4091.

**Table S1. Clinical Characteristics of the 24 Patients Included in the Microfluidic PDOTs Models**

| Variable | | Number of Patients | | | |
| --- | --- | --- | --- | --- | --- |
|  |  | TFRC^Low^ (N=12) | TFRC^High^ (N=12) | Total (N=24) | P Value |
| Age (year) | <52 | 4 (16.67%) | 2 (8.33%) | 6(25%) | 0.64 |
|  | ≥52 | 8 (33.33%) | 10 (41.67%) | 18 (75%) |  |
| Sex | Male | 11 (45.83%) | 8 (33.33%) | 19(79.17%) | 0.31 |
|  | Female | 1 (4.17%) | 4 (16.67%) | 5(20.83%) |  |
| Liver cirrhosis | No | 8 (33.33%) | 5 (20.83%) | 13(54.17%) | 0.41 |
|  | Yes | 4 (16.67%) | 7 (29.17%) | 11(45.83%) |  |
| HBsAg | Negative | 2 (8.33%) | 6 (25.00%) | 8 (33.33%) | 0.19 |
|  | Positive | 10 (41.67%) | 6 (25.00%) | 16 (66.67%) |  |
| Serum AFP (ng/mL) | <20 | 9(37.50%) | 6(25.00%) | 15(62.50%) | 0.4 |
|  | ≥20 | 3(12.50%) | 6(25.00%) | 9(37.50%) |  |
| Child-Pugh | A | 12(50.00%) | 11(45.83%) | 23(95.83%) | 0.31 |
|  | B | 0 | 1 | 1(4.17%) |  |
| Tumor size （cm） | <5 | 6(25.00%) | 4(16.67%) | 10(41.67%) | 0.68 |
|  | ≥5 | 6(25.00%) | 8(33.33%) | 14(58.33%) |  |
| Tumor number | Single | 11(45.83%) | 5(20.83%) | 16(66.67%) | 0.03 |
|  | Multiple | 1 (4.17%) | 7(29.17%) | 8(33.33%) |  |
| Tumor encapsulation | No | 9(37.50%) | 6(25.00%) | 15(62.50%) | 0.4 |
|  | Yes | 3(12.50%) | 6(25.00%) | 9(37.50%) |  |
| Tumor differentiation | I-II | 10(41.67%) | 3(12.50%) | 13(54.17%) | 0.01 |
|  | III-IV | 2(8.33%) | 9(37.50%) | 11(45.83%) |  |
| Vascular invasion | Absent | 7 (29.17%) | 4 (16.67%) | 11 (45.83%) | 0.41 |
|  | Present | 5 (20.83%) | 8 (33.33%) | 13 (54.17%) |  |
| CNLC stage | I-II | 12 (50.00%) | 6 (25.00%) | 18 (75.00%) |  |
|  | IIIa | 2 (8.33%) | 4 (16.67%) | 6 (25.00%) | 0.15 |

**Abbreviation:** TFRC, Transferrin Receptor; AFP, a-fetoprotein; CNLC, China liver cancer staging

**Table S2. Clinical characteristics of 21 patients with advanced HCC treated with an anti-PD-1-based treatment**

| Variable | | Number of Patients | | | |
| --- | --- | --- | --- | --- | --- |
|  |  | Response | | non-Response | |
|  |  | CR (n=0) | PR (n=4) | SD (n=9) | PD (n=8) |
| Age (year) | <52 | 0 | 1 | 3 | 3 |
|  | ≥52 | 0 | 3 | 6 | 5 |
| Sex | Male | 0 | 3 | 8 | 5 |
|  | Female | 0 | 1 | 1 | 3 |
| Liver cirrhosis | No | 0 | 4 | 7 | 5 |
|  | Yes | 0 | 0 | 2 | 3 |
| HBsAg | Negative | 0 | 3 | 6 | 3 |
|  | Positive | 0 | 1 | 3 | 5 |
| Serum AFP (ng/mL) | <20 | 0 | 3 | 8 | 4 |
|  | ≥20 | 0 | 1 | 1 | 4 |
| Tumor size | <5 (CM) | 0 | 2 | 5 | 7 |
|  | ≥5 (CM) | 0 | 2 | 4 | 1 |
| Tumor number | Single | 0 | 4 | 6 | 6 |
|  | Multiple | 0 | 0 | 3 | 2 |
| Vascular invasion | Absent | 0 | 2 | 4 | 5 |
|  | Present | 0 | 2 | 5 | 3 |
| CNLC stage | I-II | 0 | 1 | 2 | 0 |
|  | III-IV | 0 | 3 | 7 | 8 |
| TFRC | Low | 0 | 3 | 5 | 2 |
|  | High | 0 | 1 | 4 | 6 |
| **Abbreviation:** CR, complete response; PR, partial response; SD, stable disease; PD, progressive disease; TFRC, Transferrin Receptor; AFP, a-fetoprotein; CNLC, China liver cancer staging. | | | | | |

**Table S3. Summary of the primer sequences**

| Gene Target | Primer Direction | Primer Sequence |
| --- | --- | --- |
| TFRC | Forward | 5’-ACCATTGTCATATACCCGGTTCA-3’ |
|  | Reverse | 5’-CAATAGCCCAAGTAGCCAATCAT-3’ |
| CXCL14 | Forward | 5’-CGCTACAGCGACGTGAAGAA-3’ |
|  | Reverse | 5’-GTTCCAGGCGTTGTACCAC-3’ |
| CD22 | Forward | 5’-AGAACACCTCGAAGTTTGATGG-3’ |
|  | Reverse | 5’-CCAGGAATTGCACCCTTTTCTG-3’ |
| FLT3 | Forward | 5’-AGGGACAGTGTACGAAGCTG-3’ |
|  | Reverse | 5’- GCTGTGCTTAAAGACCCAGAG-3’ |
| GAPDH | Forward | 5’-TGATGACATCAAGAAGGTGGTGAAG-3’ |
|  | Reverse | 5’-TCCTTGGAGGCCATGTGGGCCAT-3’ |

**Abbreviation:** TFRC, Transferrin Receptor; GAPDH, Glyceraldehyde 3-Phosphate Dehydrogenase; CXCL14, C-X-C Motif Chemokine Ligand 14; FLT3, Fms Related Receptor Tyrosine Kinase 3.

**Table S4. Summary of Antibodies**

| Name | Supplier | Cat no. | Clone no. |
| --- | --- | --- | --- |
| Rabbit anti-TFRC | Abcam | ab214039 | EPR20584 |
| Rabbit anti-GAPDH | Cell Signaling Technology | 5174 | D16H11 |
| Rabbit anti-CXCL14 | Abcam | ab137541 | Polyclonal antibody |
| Rabbit anti-CD4 | Abcam | ab133616 | EPR6855 |
| Rabbit anti-CD8 | Abcam | ab245118 | EPR22483-288 |
| Rabbit anti-CD8 | Abcam | ab209775 | EPR20305 |
| Rabbit anti-CD19 | Abcam | ab245235 | EPR23174-145 |
| Rabbit anti-P-VEGFR2 | Abcam | ab38473 | Polyclonal antibody |
| Rabbit anti-VEGFR2 | Servicebio | GB11190 | Polyclonal antibody |
| Rabbit anti-P-FGFR1 | Cell Signaling Technology | 52928 | D4X3D |
| Rabbit anti-FGFR1 | Cell Signaling Technology | Tyr766 | 2544 |
| Rabbit anti-P-PDGFRβ | Cell Signaling Technology | 3161 | Tyr751 |
| Rabbit anti-PDGFRβ | Cell Signaling Technology | 3169 | 28E1 |
| Rabbit anti-p-AKT | Cell Signaling Technology | 4060 | Ser473 |
| Rabbit anti-AKT | Servicebio | GB111114 | Polyclonal antibody |
| Rabbit anti-HIF1α | Abcam | ab228649 | Polyclonal antibody |
| Goat Anti-Rabbit IgG H&L | Servicebio | G1213-100UL | \ |
| Alexa Fluor® 488 Anti-CD45 antibody | Abcam | ab197730 | F10-89-4 |
| Alexa Fluor® 488 Anti-CD8 alpha antibody | Abcam | ab196462 | EP1150Y |

**Abbreviation:** TFRC, Transferrin Receptor; GAPDH, Glyceraldehyde 3-Phosphate Dehydrogenase; CXCL14, C-X-C Motif Chemokine Ligand 14; P-VEGFR2, Phosphorylated Vascular Endothelial Growth Factor Receptor 2; VEGFR2, Vascular Endothelial Growth Factor Receptor 2; P-FGFR1, Phosphorylated Fibroblast Growth Factor Receptor 1; FGFR1, Fibroblast Growth Factor Receptor 1; P-PDGFRβ, Phosphorylated Platelet-Derived Growth Factor Receptor Beta; PDGFRβ, Platelet-Derived Growth Factor Receptor Beta; p-AKT, Phosphorylated AKT Serine/Threonine Kinase; AKT, AKT Serine/Threonine Kinase; HIF1α, Hypoxia-Inducible Factor 1 Alpha.

**Table S5. Comparative Tumor Volume Measurements in Response to Various Treatments as Depicted in Figure 1E**

| Group | No. | L(mm) | W(mm) | V(mm^3^) |
| --- | --- | --- | --- | --- |
| Anlotinib+anti-PD-1 | 14 | 5.77 | 5.2 | 78.01 |
|  | 21 | 7.9 | 5.2 | 106.81 |
|  | 13 | 10.8 | 6.5 | 228.15 |
|  | 20 | 7.25 | 5.38 | 104.92 |
|  | 9 | 8.22 | 5.5 | 124.33 |
|  | 33 | 9.23 | 6.3 | 183.17 |
| Anlotinib | 3 | 10.8 | 8.22 | 364.87 |
|  | 28 | 12.52 | 7.55 | 356.84 |
|  | 35 | 11.07 | 8.67 | 416.06 |
|  | 6 | 11.8 | 9.26 | 505.91 |
|  | 22 | 6.7 | 6.3 | 132.96 |
|  | 8 | 11.08 | 6.44 | 229.76 |
| Anti-PD-1 | 15 | 11.54 | 7.01 | 283.54 |
|  | 1 | 11.6 | 6.66 | 257.26 |
|  | 31 | 12.15 | 9.34 | 529.96 |
|  | 19 | 10.44 | 7.75 | 313.53 |
|  | 32 | 10.93 | 6.26 | 214.16 |
|  | 25 | 15.8 | 9.47 | 708.48 |
| Control | 36 | 12.71 | 9.15 | 532.06 |
|  | 17 | 16.64 | 11.47 | 1094.59 |
|  | 34 | 17.34 | 12.23 | 1296.80 |
|  | 2 | 13.4 | 9.35 | 585.73 |
|  | 24 | 14.4 | 9.96 | 714.25 |
|  | 12 | 13 | 10.84 | 763.79 |

**Table S6. Comparative Tumor Volume Measurements in Response to Various Treatments as Depicted in Figure S2B**

| Group | No. | L(mm) | W(mm) | V(mm^3^) |
| --- | --- | --- | --- | --- |
| Anlotinib+anti-PD-1 | 12707 | 10.25 | 7.55 | 292.14 |
|  | 12706 | 7.16 | 6.7 | 160.71 |
|  | 12710 | 6.5 | 6.47 | 136.05 |
|  | 12708 | 7.3 | 6 | 131.40 |
|  | 12709 | 6.5 | 5.5 | 98.31 |
|  | 12711 | 3.5 | 3 | 15.75 |
| Anlotinib | 12726 | 10.95 | 10.33 | 584.23 |
|  | 12704 | 13.3 | 9.18 | 560.41 |
|  | 12703 | 13 | 8.4 | 458.64 |
|  | 12728 | 11.77 | 8.88 | 464.06 |
|  | 12723 | 9.85 | 7.77 | 297.34 |
|  | 12722 | 9.06 | 8.07 | 295.02 |
| Anti-PD-1 | 12719 | 12.76 | 10.31 | 678.17 |
|  | 12729 | 11.7 | 8.9 | 463.38 |
|  | 12737 | 11.42 | 8.45 | 407.71 |
|  | 12713 | 11.2 | 8.4 | 395.14 |
|  | 12716 | 10.4 | 8.6 | 384.59 |
|  | 12705 | 10.55 | 8.21 | 355.56 |
| Control | 12702 | 17.6 | 12.66 | 1410.43 |
|  | 12736 | 14 | 12.9 | 1164.87 |
|  | 12734 | 14.03 | 11.44 | 918.08 |
|  | 12731 | 14.38 | 10.27 | 758.35 |
|  | 12718 | 14.77 | 11.01 | 895.21 |
|  | 12712 | 17.4 | 10 | 870.00 |

**Table S7. Comparative Tumor Volume Measurements in Response to Various Treatments as Depicted in Figure 5B**

| Group | No. | L(mm) | W(mm) | V(mm^3^) |
| --- | --- | --- | --- | --- |
| shTFRC+anti-PD-1 | 12300 | 17.36 | 12.92 | 1448.92 |
|  | 12304 | 20.79 | 11.8 | 1447.40 |
|  | 12300 | 16.17 | 12.83 | 1330.86 |
|  | 12302 | 14.64 | 13.33 | 1300.68 |
|  | 12308 | 17.28 | 11.68 | 1178.69 |
|  | 12307 | 11.39 | 6.94 | 274.29 |
| shTFRC | 12301 | 21.55 | 18.11 | 3533.90 |
|  | 12303 | 27.93 | 15.84 | 3503.90 |
|  | 12304 | 21.99 | 14.94 | 2454.12 |
|  | 12301 | 22.92 | 14.6 | 2442.81 |
|  | 12310 | 20.21 | 14.47 | 2115.79 |
|  | 12304 | 21.78 | 12.9 | 1812.20 |
| Anti-PD-1 | 12310 | 24.34 | 17.04 | 3533.70 |
|  | 12309 | 21.74 | 15.85 | 2730.79 |
|  | 12302 | 20.5 | 16.1 | 2656.90 |
|  | 12304 | 20.38 | 15.33 | 2394.74 |
|  | 12306 | 20.17 | 14.71 | 2182.23 |
|  | 12305 | 16.73 | 15.47 | 2001.92 |
| Control | 12302 | 28.91 | 17.65 | 4503.06 |
|  | 12307 | 21.11 | 20.43 | 4405.50 |
|  | 12303 | 25.89 | 18.14 | 4259.68 |
|  | 12302 | 25.57 | 17.82 | 4059.91 |
|  | 12312 | 25.05 | 17.79 | 3963.96 |
|  | 12308 | 26.53 | 17.05 | 3856.17 |

**Supplement Figure Legend**

**Figure S1.** Immune cell profiling of PDOTS. Representative dot plot images of the immune cell analysis. Singlets, live cells, and whole immune cells (leukocytes) were sequentially gated. Leukocytes were divided into myeloid cells and the T-cell populations.

**Figure S2.** The anti-tumor effects of combined anti-PD-1 and anlotinib treatment in H22 tumors. (A) Representative image of orthotopic tumors from BALB/C mice administered the treatments (n = 6 mice per group). (B) H22 tumor volumes and weights are shown in the right panel. (C) The line graphs illustrate the changes in body weight of C57BL/6 and BALB/c mice throughout the course of treatment, serving as an indicator of overall treatment tolerability. (D-E) Representative H&E and Tunel images from the primary tumor of BALB/C mice. T, tumor; N, necrosis. *, P < 0.05; **, P < 0.01; ***, P < 0.001; ns, not significant.

**Figure S3.** Differential Distribution of CD8^+^ and CD4^+^ T Cells in Treated H22 Tumors and t-SNE Analysis of CD4^+^ T Cell Subpopulations. (A) Representative images and bar plots illustrating the number of CD8^+^ and CD4^+^ T cells in H22 tumors from mice that received the indicated treatments, Scale bar: 100 µm. (B) Representative t-SNE plots of the CD4^+^ T cell subpopulation distribution. The C47 subgroup accounts for 81.38% of the total CD4^+^ T cells, while the C48 (11.12%) and C49 (7.5%) subpopulations account for 18.62% of the total CD4^+^ T cells. *, P < 0.05; **, P < 0.01; ***, P < 0.001; ns, not significant.

**Figure S4.** RNA-seq analysis of tumor treated with combination therapy versus anti-PD-1. (A) GO and KEGG enrichment analysis based on the genes that were differentially expressed after treatment with anlotinib plus an anti-PD-1 antibody. (B) GSEA analysis of the differentially expressed genes induced by treatment with anlotinib plus an anti-PD-1 antibody. Shown are three of the top ten most positively regulated “Hallmark signatures”. (C) Immune microenvironment score analysis of different treatment groups performed using xCELL algorithms. (D-E) Transcriptomic profiling of immune checkpoint markers in the HCC model mice that received different treatments. Bar graph showing normalized expression levels of TIGIT, PD-1, LAG3, CTLA4, PD-L1, and TIM3 across the control, anlotinib, anti-PD-1, and combination therapy groups. *, P < 0.05; **, P < 0.01; ***, P < 0.001; ns, not significant. (F) The bar chart quantifies the average expression levels of HIF-1α mRNA, standardized as FPKM (Fragments Per Kilobase of transcript per Million mapped reads).

**Figure S5.** Anlotinib does not inhibit CD22 or FLT3 mRNA expression in HCC cell lines. QRT-PCR analysis of the CD22 and FLT3 mRNA levels in PLC/PRF/5 and HepG2 cells treated with increasing concentrations of anlotinib (0–20 µmol) for 24 h. *, P < 0.05; ns, not significant.

**Figure S6.** Anlotinib inhibits TFRC by affecting the VEGF/AKT/HIF-1α signaling pathway. (A) Densitometry analysis of three experiments representative of the data shown in Figure 4E. (B-C) Densitometry analysis of three experiments representative of the data shown in Figure 4F. *, P < 0.05; **, P < 0.01; ***, P < 0.001; ns, not significant.

**Figure S7.** Exploring TFRC and CXCL14 Expression Impact on HCC Immune Microenvironment and CD8^+^ T Cell Dynamics. (A) A box plot of the cytotoxic cells and CD8^+^ T cells in the low-and high-TFRC groups. The median TFRC expression was used as the cut-off to divide HCC patients into low- and high-TFRC groups. (B-C) The efficiency of TFRC knockdown was validated by qRT-PCR and Western blotting. (D) Quantitative analysis of CD8^+^ T cell migration towards tumor cells and the subsequent tumor cell viability assay. Activated CD8^+^ T cells were placed in the upper chamber of a migration setup, with PLC/PRF/5 HCC cells treated with either vehicle, TFRC knockdown, or anlotinib in the lower chamber. After a 48-hour co-culture period, the migrated CD8^+^ T cells were quantified. The viability of the HCC cells was assessed by crystal violet staining. (E) The box plot of the T cells, CD8^+^ T cells, cytotoxic cells, T helper cells and Treg cells between the low- and high-CXCL14 groups. The median CXCL14 expression was used as the cut-off to divide HCC patients into low- and high-CXCL14 groups. (F) The immune microenvironment score of different treatment groups determined using XCELL algorithms. *, P < 0.05; **, P < 0.01; ***, P < 0.001; ns, not significant.

**Figure S8.** Effect of Anlotinib on CXCL14 Expression and Secretion in Hepatocellular Carcinoma. (A) QRT-PCR analysis of the CXCL14 mRNA levels in PLC/PRF/5 and HepG2 cells treated with increasing concentrations of anlotinib (0–20 µmol) for 24 h. (B) ELISA analysis of the CXCL14 mRNA levels in PLC/PRF/5 and HepG2 cells treated with increasing concentrations of anlotinib (0–20 µmol) for 24 h. (C) ELISA showing that anlotinib increases CXCL14 secretion by HCC PDOTS in vitro after 48 hours (n=7). *, P < 0.05; **, P < 0.01; ***, P < 0.001; ns, not significant.

**Figure S9.** Anlotinib enhances CXCL14 expression through the downregulation of TFRC. (A-B) Quantitative real-time PCR (qRT-PCR) analysis of the CXCL14 transcription levels. Each bar represents the relative mRNA expression normalized to the siNC+Vehicle group. Enzyme-Linked Immunosorbent Assay (ELISA)-based quantification of the CXCL14 concentration in cell culture supernatants. Data are presented in pg/ml. (C) Western Blot analysis illustrating the CXCL14 protein expression, where each band represents the relative protein abundance compared to the siNC+Vehicle group. These findings highlight the variations in CXCL14 expression across different treatments. Statistical significance is indicated as follows: *, P < 0.05; **, P < 0.01; ***, P < 0.001; ns, not significant.

**Figure S10.** Comparative Analysis of Tumor Killing Index in High versus Low TFRC Expression Groups Under Anti-PD1 and Anlotinib Therapies. A bar graph illustrating the tumor killing index percentages for both the high TFRC and low TFRC groups upon treatment with either anti-PD1 alone or in combination with anlotinib. Each dot within the bars represents data from an individual patient. *, P < 0.05; **, P < 0.01; ***, P < 0.001; ns, not significant.

**Figure S1**


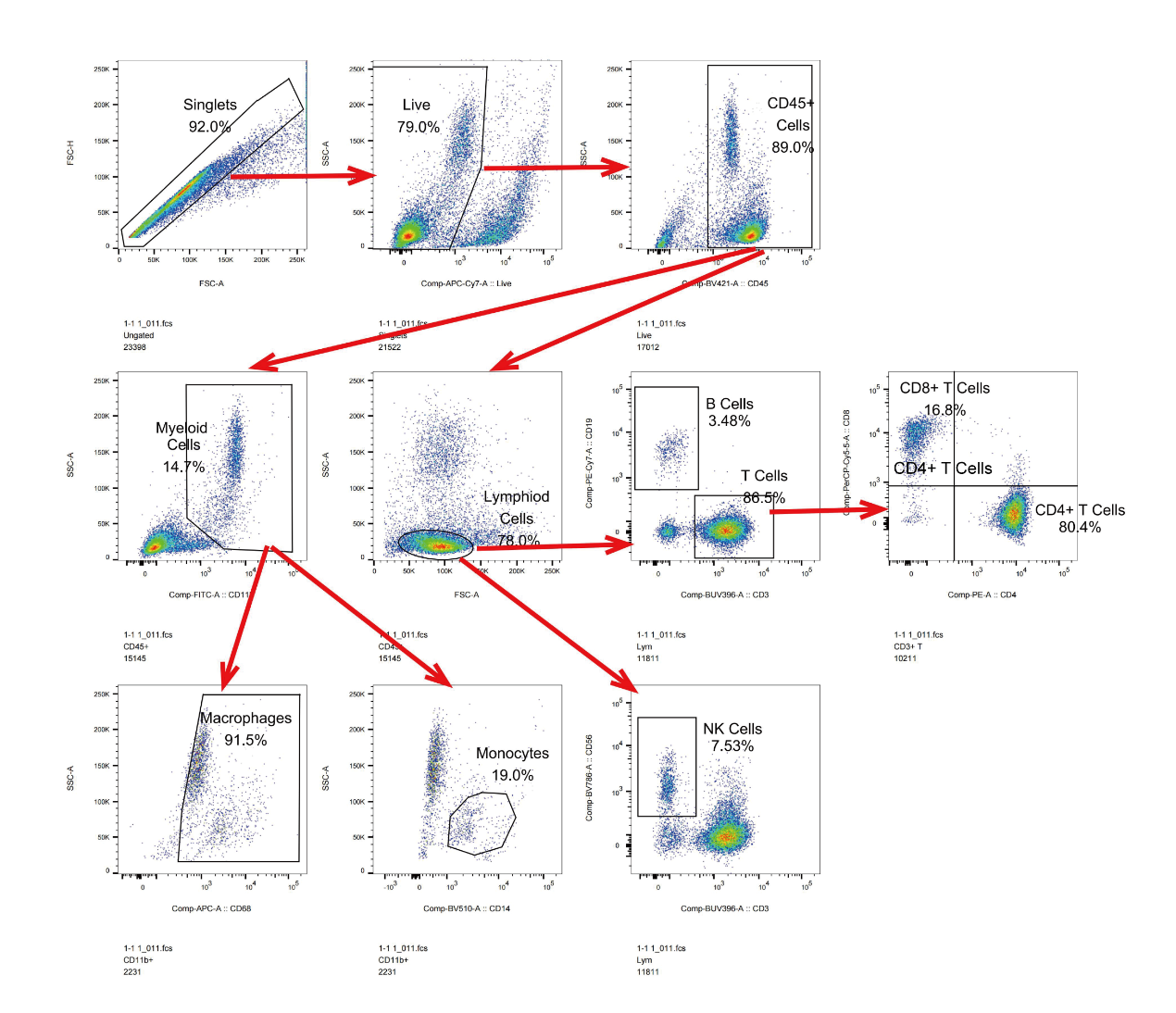


**Figure S2**


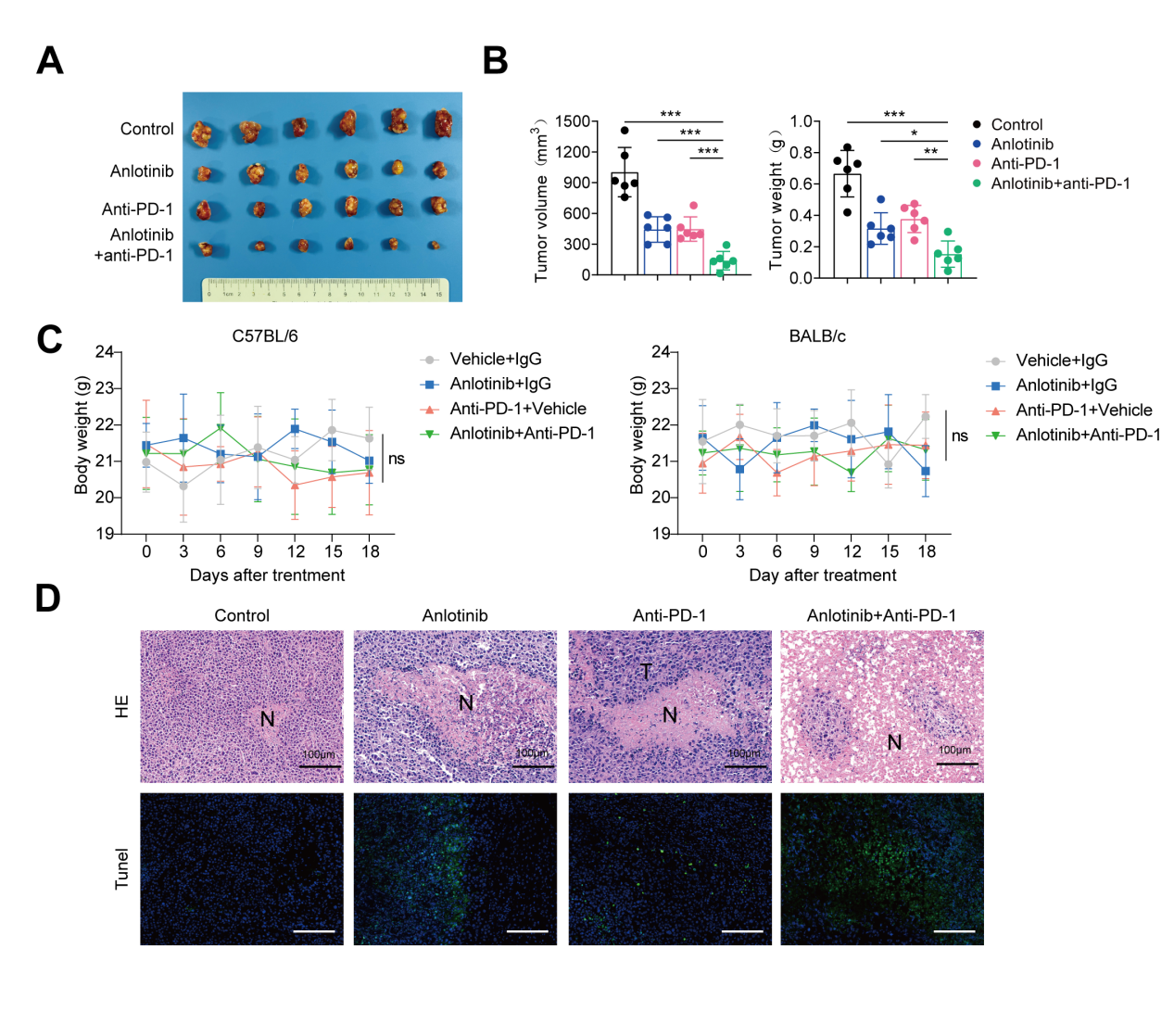


**Figure S3**


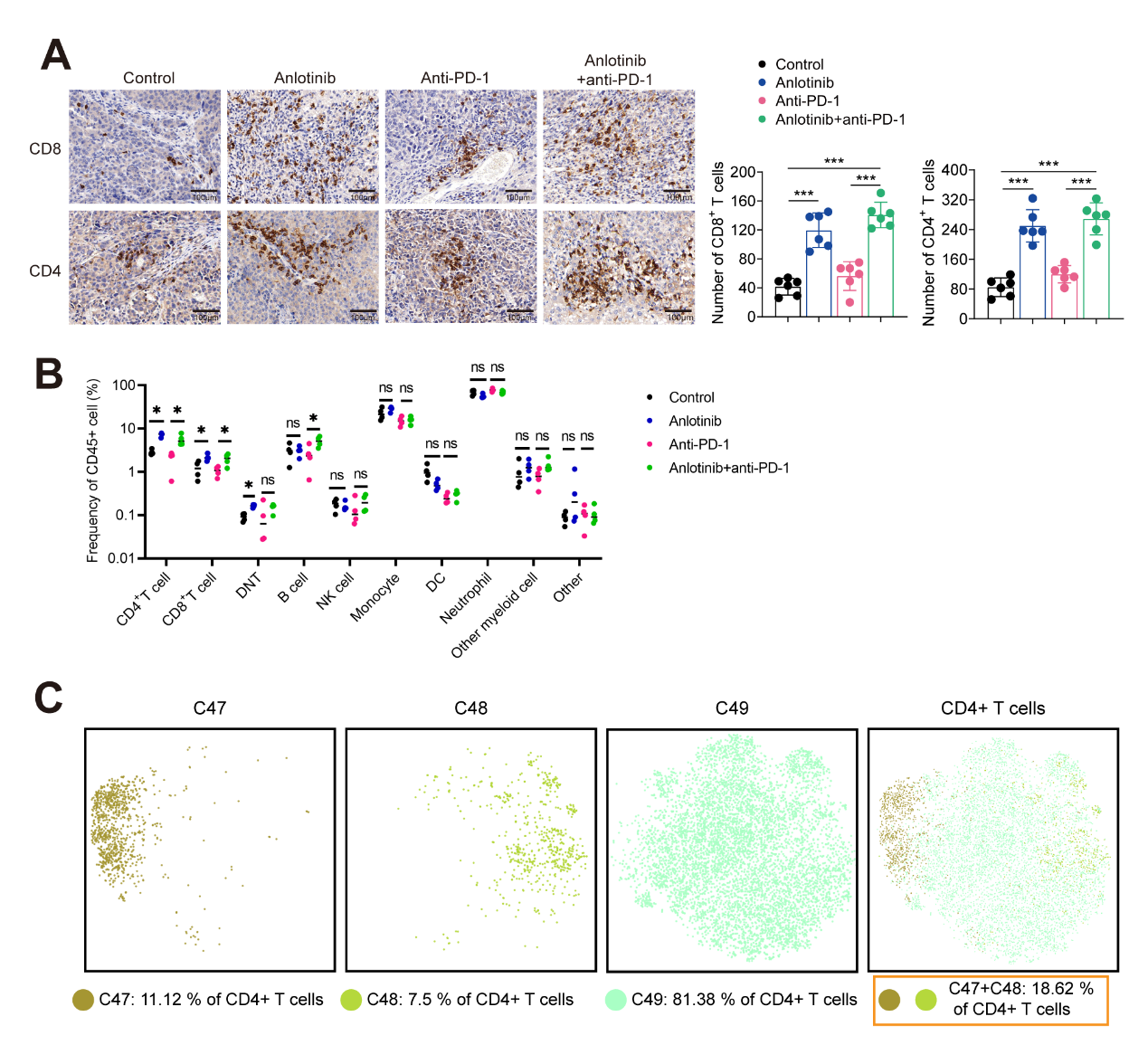


**Figure S4**


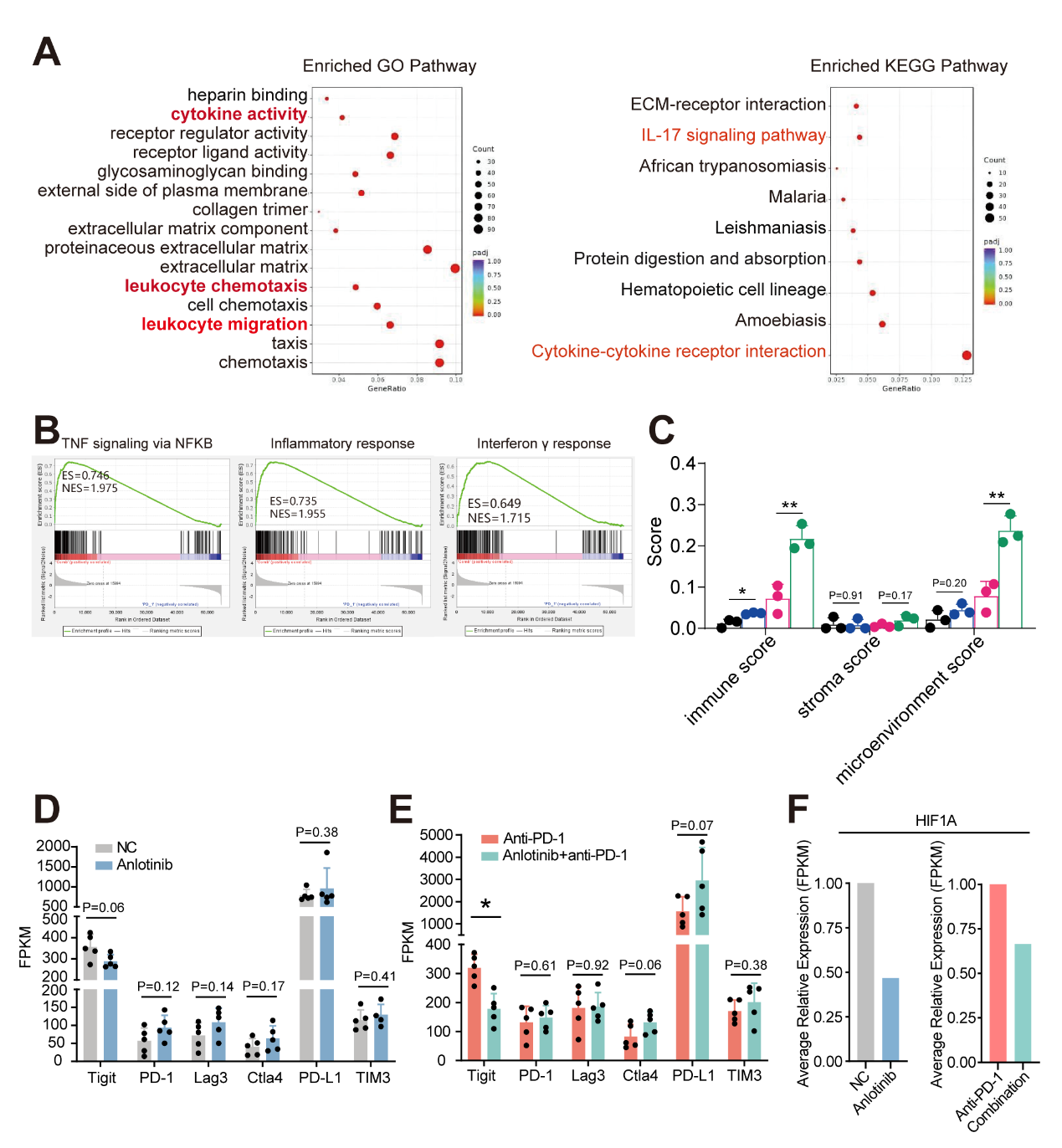


**Figure S5**


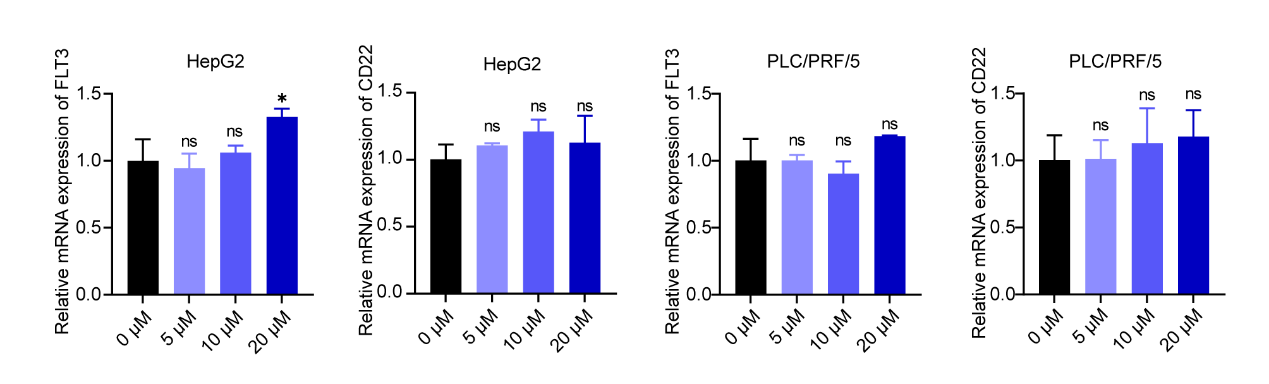


**Figure S6**


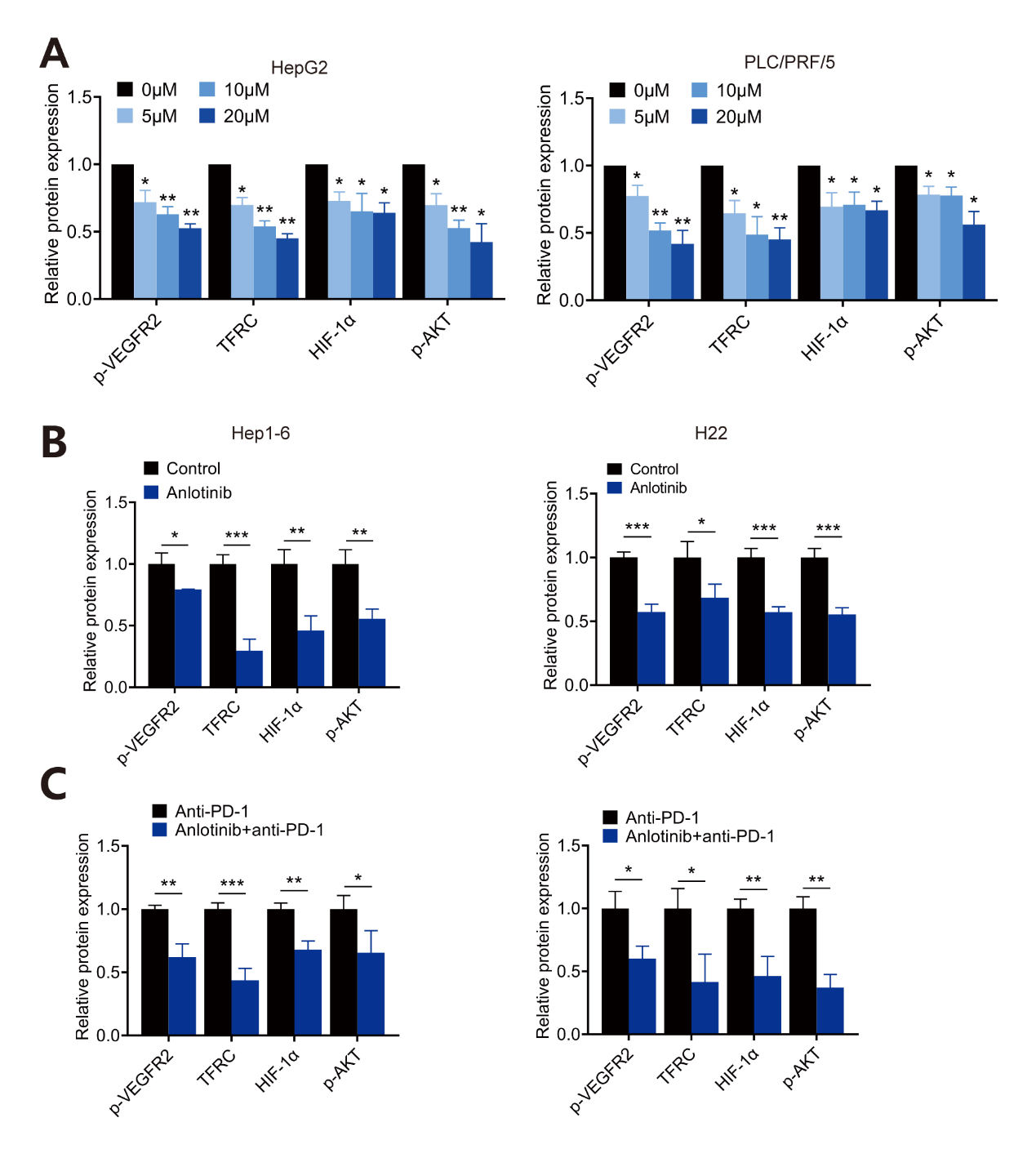


**Figure S7**


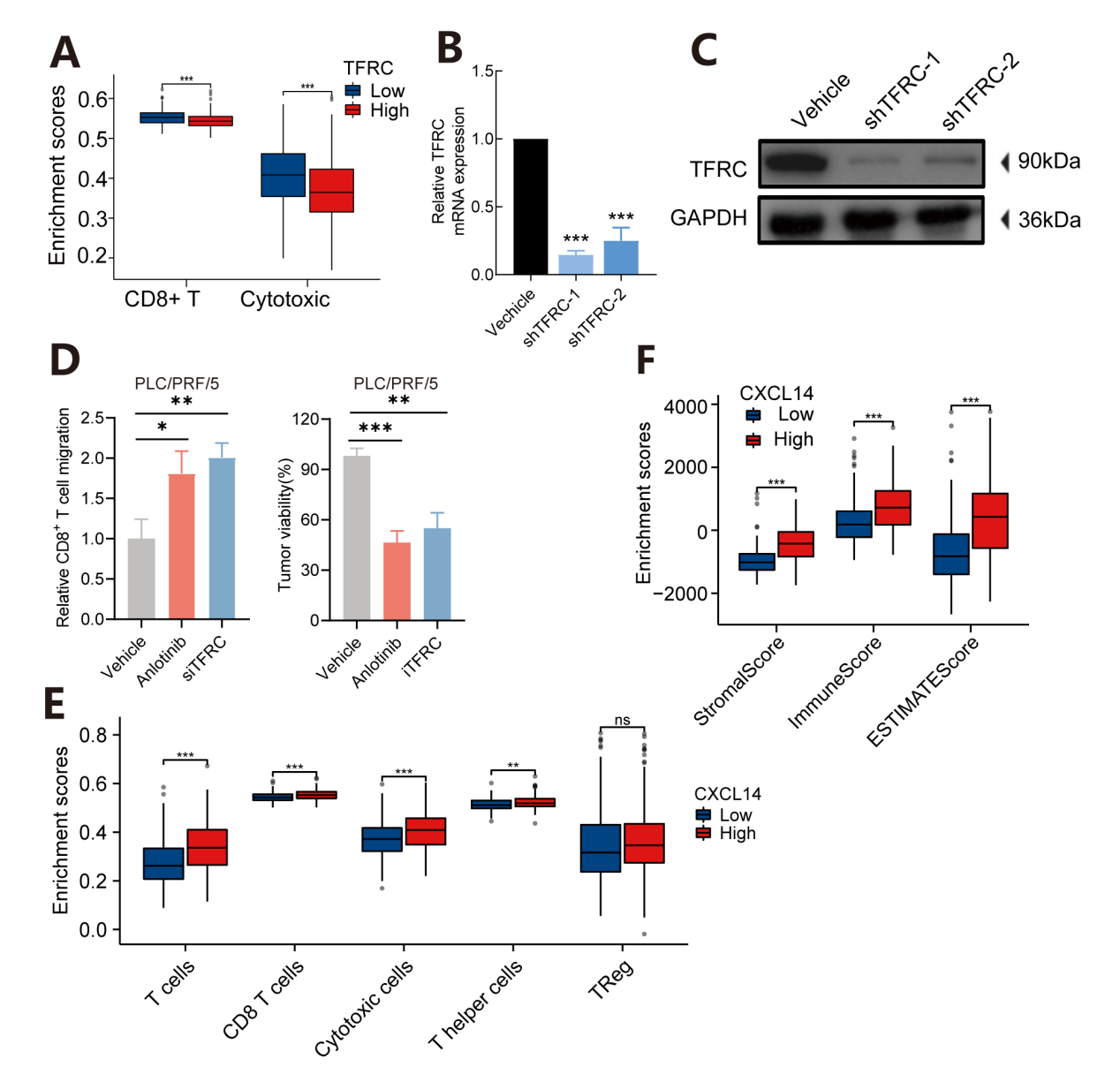


**Figure S8**


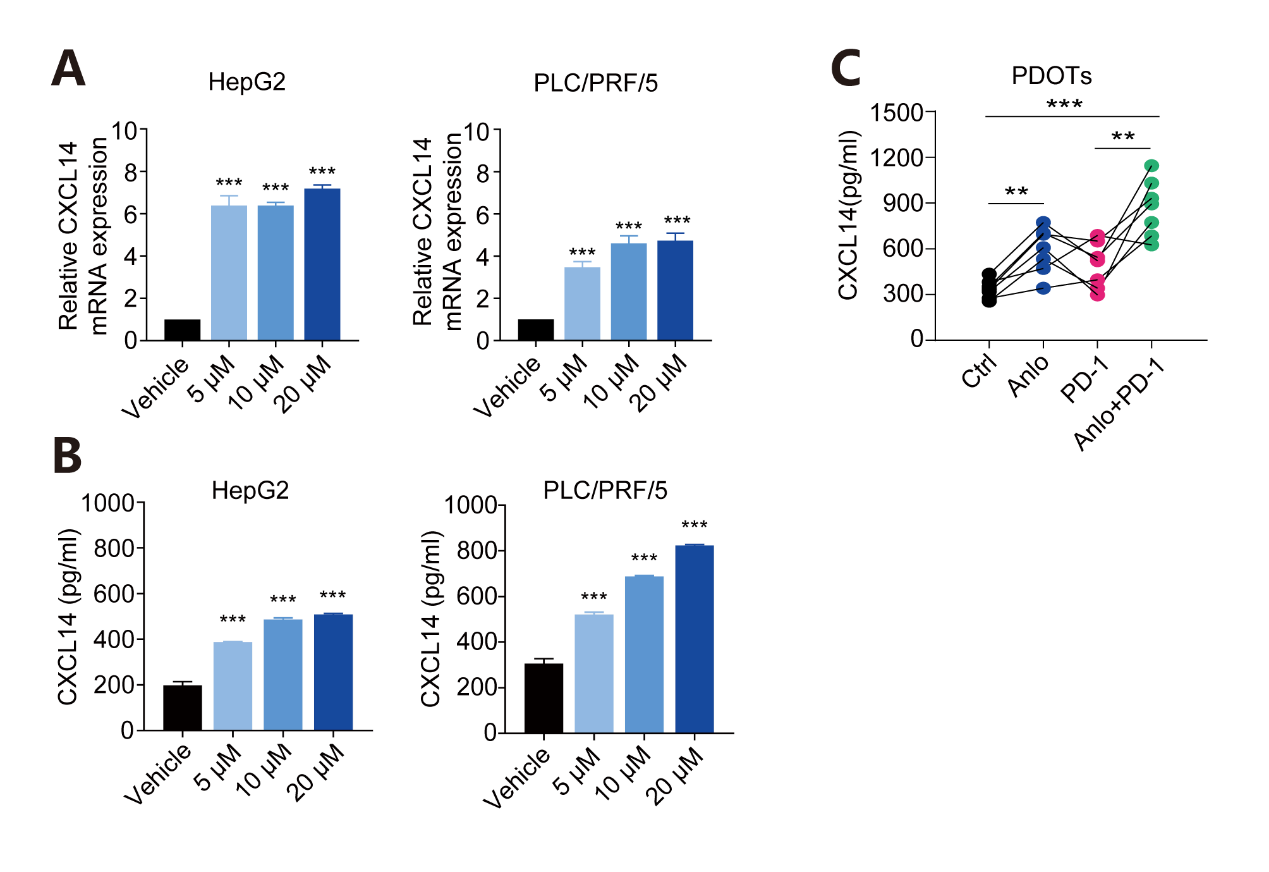


**Figure S9**


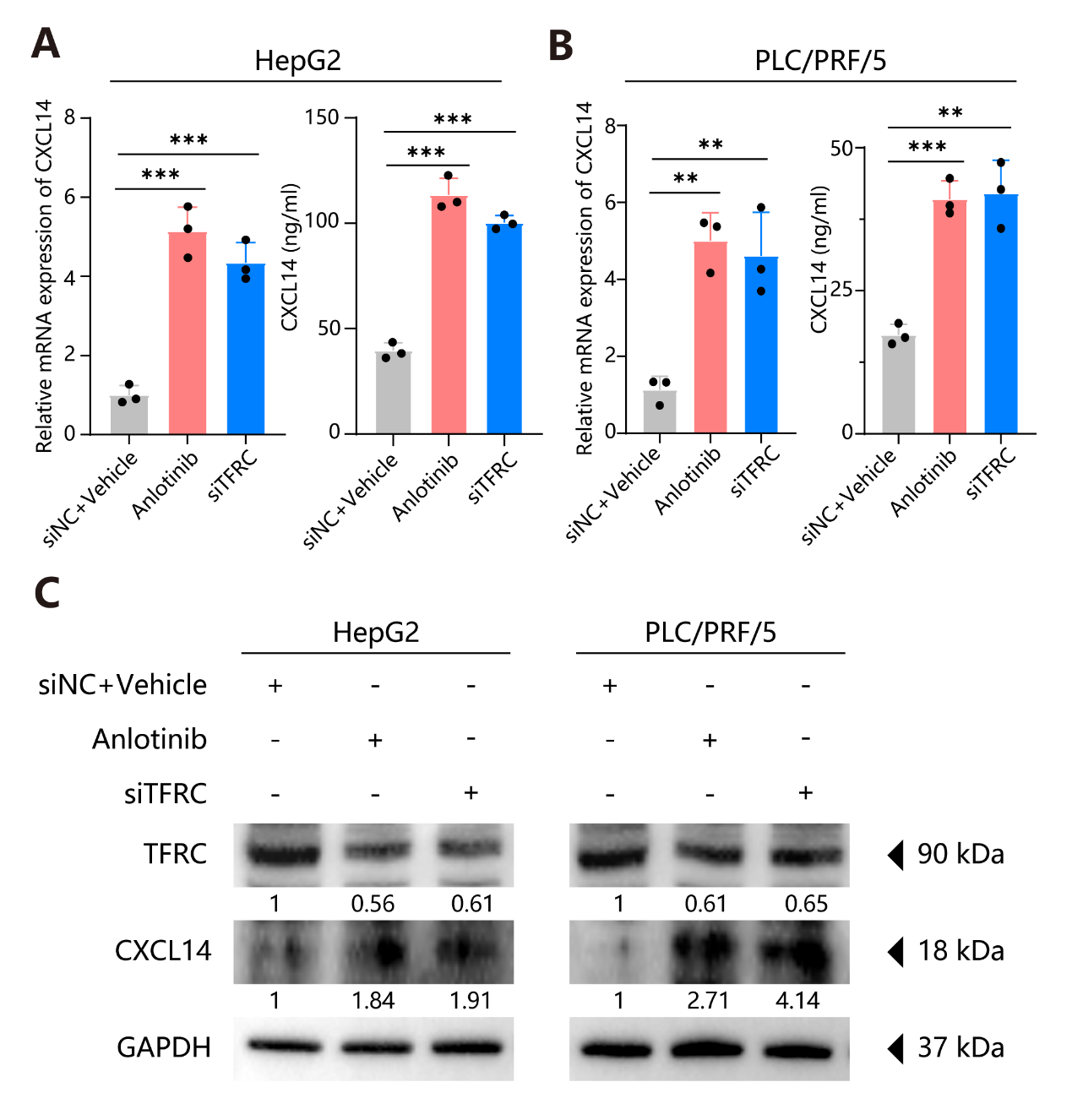


**Figure S10**


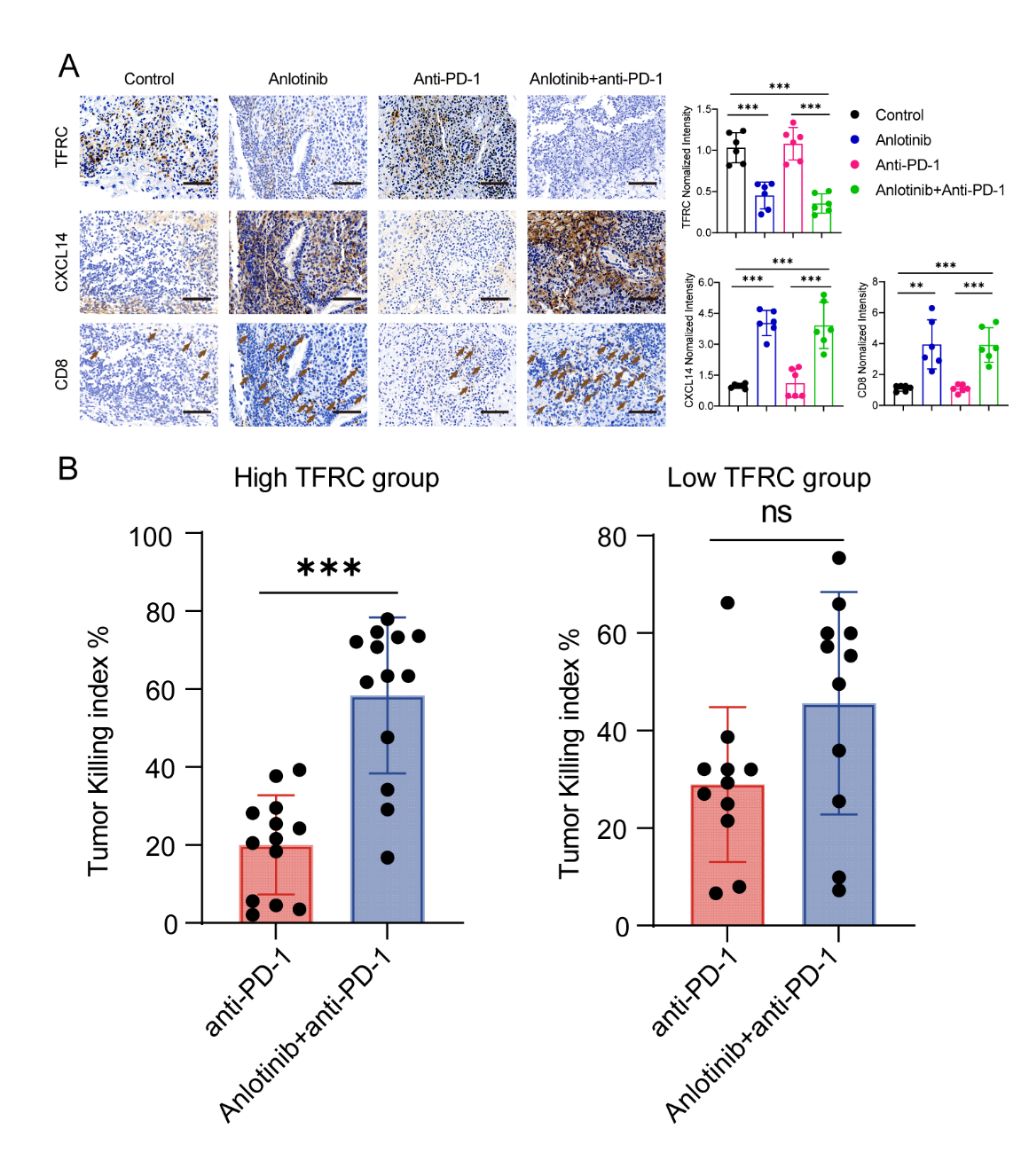

Supplement: Supplementary file 1 — Supporting Information [file CTM2-14-e1738-s001.docx]
